# Allele specific repair of splicing mutations in cystic fibrosis through AsCas12a genome editing

**DOI:** 10.1038/s41467-019-11454-9

**Published:** 2019-08-07

**Authors:** Giulia Maule, Antonio Casini, Claudia Montagna, Anabela S. Ramalho, Kris De Boeck, Zeger Debyser, Marianne S. Carlon, Gianluca Petris, Anna Cereseto

**Affiliations:** 1 Centre for Integrative Biology (CIBIO), University of Trento, Via Sommarive 9, 38123 Trento, Italy; 2 Department of Development and Regeneration, CF Centre, Woman and Child, KU Leuven, Herestraat 49, Leuven, 3000 Belgium; 3 Pediatric Pulmonology, Department of Pediatrics, University Hospital Leuven, Herestraat 49, Leuven, 3000 Belgium; 4 Laboratory for Molecular Virology and Drug Discovery, Department of Pharmaceutical and Pharmacological Sciences, KU Leuven, Herestraat 49, Leuven, 3000 Belgium; 5 Present Address: Medical Research Council Laboratory of Molecular Biology, Cambridge Biomedical Campus, Francis Crick Avenue, Cambridge, CB2 0QH UK

**Keywords:** Cystic fibrosis, CRISPR-Cas9 genome editing

## Abstract

Cystic fibrosis (CF) is an autosomal recessive disease caused by mutations in the *CFTR* gene. The 3272–26A>G and 3849+10kbC>T *CFTR* mutations alter the correct splicing of the *CFTR* gene, generating new acceptor and donor splice sites respectively. Here we develop a genome editing approach to permanently correct these genetic defects, using a single crRNA and the *Acidaminococcus sp. BV3L6*, AsCas12a. This genetic repair strategy is highly precise, showing very strong discrimination between the wild-type and mutant sequence and a complete absence of detectable off-targets. The efficacy of this gene correction strategy is verified in intestinal organoids and airway epithelial cells derived from CF patients carrying the 3272–26A>G or 3849+10kbC>T mutations, showing efficient repair and complete functional recovery of the CFTR channel. These results demonstrate that allele-specific genome editing with AsCas12a can correct aberrant *CFTR* splicing mutations, paving the way for a permanent splicing correction in genetic diseases.

## Abstract (web summary)

Cystic fibrosis is caused by mutations in the CFTR chloride channel. Here, the authors develop a gene therapy approach using the programmable nuclease AsCas12a to correct a splicing mutation in CFTR, and show efficient repair of the mutation and recovery of CFTR function in patient-derived organoids and airway epithelial cells.

## Introduction

Cystic fibrosis (CF) is a lethal autosomal recessive inherited disorder with an approximate frequency of 1 in 2500 births. CF is linked to mutations in the cystic fibrosis transmembrane conductance regulator (*CFTR*) gene^1^, which encodes a chloride/bicarbonate channel expressed in the apical membrane of epithelial cells. The lack of ion conductance across the membrane of these cells leads to impaired ion and liquid homeostasis, generating a multi-organ disorder. The primary cause of mortality in CF patients is bacterial infections of the airways, provoking chronic lung disease and ultimately respiratory failure^2^. Current CF treatments are not curative and limited to the reduction of clinical symptoms including intestinal-airway blockages and chronic bacterial infections. Recent therapeutic advances were obtained in CF treatments through the development of CFTR correctors and potentiators^3,4^, which however target exclusively few types of mutations including the highly recurrent ΔF508.

In search for a cure for CF, several gene therapy approaches have been explored^5^, mostly based on *CFTR* cDNA gene addition through viral or non-viral vectors^6,7^. Despite promising results obtained in the respiratory tract of animal models^8–10^ and advancements in gene therapy clinical trials^11–14^, curative goals were hampered mainly by low expression levels of the delivered *CFTR*.

The recent advances in genome editing, with the development of precise and efficient CRISPR-nucleases, have highly accelerated the progress of gene correction for genetic diseases, including CF^15^. Following initial discovery of the *Streptococcus pyogenes* Cas9 (SpCas9), several additional CRISPR-nucleases have been discovered with different functional, mechanistic and structural features, expanding the genome editing tool-box^16^. Among these, AsCas12a has been widely used for its different PAM requirements and a natural very high specificity^17–20^.

By means of genome editing, in contrast to the classical gene addition strategies, the correction of the mutated *CFTR* holds the promise to restore physiological levels of CFTR expression and function. In CF cellular models *CFTR* genetic repair was obtained through strategies exploiting the cellular homology-directed repair (HDR) pathway^21,22^. Nevertheless, the HDR pathway is not highly active in human cells, thus strongly limiting the clinical efficacy of this application^23,24^. Alternatively, genetic modification by non-homologous end joining (NHEJ) is more efficient and does not require the delivery of a donor DNA template. CRISPR-nucleases have been successfully used to induce NHEJ to knockout genes or genomic regulatory elements^25,26^. This includes permanent repair of splicing defects in *CFTR* minigene models^27^ as an alternative to the transient and inefficient use of oligonucleotides or spliceosome-mediated RNA trans-splicing (SMaRT) strategies^28,29^.

Here we develop a genome editing strategy to repair 3272-26A>G (c.3140-26A>G) and 3849+10kbC>T (c.3718-2477C>T) *CFTR* mutations. The 3242-26A>G is a point mutation that creates a new acceptor splice site causing the abnormal inclusion of 25 nucleotides within exon 20^30,31^. The resulting mRNA contains a frameshift in *CFTR*, producing a premature termination codon and consequent expression of a truncated non-functional CFTR protein. The 3849+10kbC>T mutation creates a novel donor splice site inside intron 22 of the *CFTR* gene, leading to the insertion of the new cryptic exon of 84 nucleotides, which results in an in-frame stop codon and consequent production of a truncated non-functional CFTR protein^32,33^.

In this study, we harness the AsCas12a nuclease with a single CRISPR RNA (crRNA) to repair the *CFTR* 3272–26A>G and 3849+10kbC>T splicing defects in different cell types including primary CF patients’ airway epithelial cells and intestinal organoids. The genome editing strategy that we develop is highly specific, as demonstrated by a preserved second allele and complete absence of off-target cleavages. CFTR functional recovery by the AsCas12a single crRNA strategy is validated in intestinal organoids derived from CF patients carrying the 3272-26A>G or the 3849+ 10kbC>T mutations, thus highlighting the power of this approach for the permanent correction of genetic diseases caused by deep intronic splicing mutations.

## Results

### Splicing correction of a 3272-26A>G minigene model

Minigenes are modeled exon–intron genetic constructs that are useful for studying RNA splicing regulation^34–36^. These constructs are commonly used to study specific cis-acting elements and their binding factors involved in constitutive or alternative splicing regulation^37^.

**Fig. 1:**
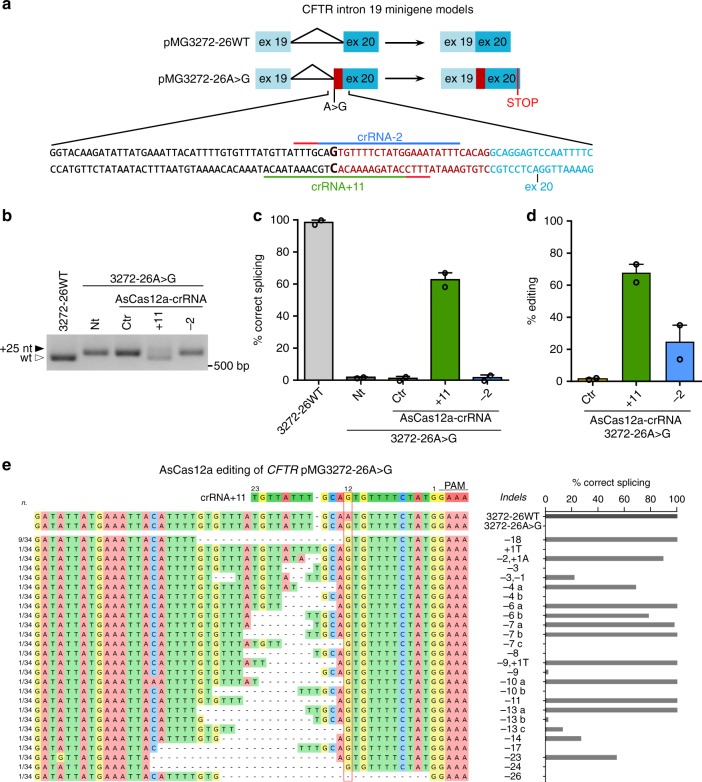
Splicing correction in *CFTR* 3272-26A>G minigene model by AsCas12a editing. **a** Scheme of the *CFTR* minigenes containing a sequence (~1.3 kb) corresponding to the *CFTR* region extending from exon 19 to 20 either wild type (pMG3272-26WT) or 3272-26A>G mutated (pMG3272-26A>G). Exons are shown as boxes, introns as lines; the expected spliced transcripts are represented on the right according to the presence or absence of the 3272-26A>G mutation. The lower panel shows nucleotide sequence and intron–exon boundaries nearby the 3272-26A>G mutation (labeled in bold) and the target AsCas12a-crRNAs position (underlined, with the PAM in red). **b** Splicing pattern analyzed by RT-PCR in HEK293/pMG3272–26A>G cells following treatments with AsCas12a-crRNA control (Ctr) or specific for the 3272-26A>G mutation (+11 and −2). Black-solid arrow indicates aberrant splicing, white-empty arrow indicates correct splicing. Representative data of *n* = 2 independent experiments. **c** Percentages of correct splicing measured by densitometry and **d** editing efficiency analyzed by TIDE in cells treated as in **b**. Data are means ± SEM from *n* = 2 independent experiments. **e** Indels triggered by AsCas12a-crRNA+11. The 3272-26A>G locus from cells edited by crRNA +11 were amplified, cloned in the minigene backbone and Sanger sequenced (34 different clones, left panel), or analyzed as in **b** for produced splicing pattern (right panel and Supplementary Fig. 3b). pMG3272-26WT and pMG3272-26A>G were used as references

We generated minigene models to mimic the splicing pattern of the *CFTR* gene corresponding to the region encompassing part of exon 18, full length exons 19 and 20, and intron 19 (legacy name: exon 16, 17a, 17b, and intron 17a), either wild-type (pMG3272-26WT) or carrying the 3272-26A>G mutation (pMG3272-26A>G) (Fig. 1a). The altered or correct splicing pattern produced by the mutated or wild-type minigenes, respectively, was evaluated by RT-PCR and sequencing analyses in transfected HEK293T cells (Supplementary Fig. 1a, b)^30^.

We then designed single guide RNA (sgRNA)^16^ and crRNA, for SpCas9 and AsCas12a, respectively (Supplementary Data 1), to generate single or double cleavages to obtain either isolated indels or deletions within intron 19, near the 3272-26A>G mutation. The splicing pattern of the pMG3272-26A>G was evaluated after its transient co-transfection with the sgRNAs/crRNAs in combination with either SpCas9 or AsCas12a (Supplementary Fig. 2a, b). We observed increased levels of the correct splicing product by using either SpCas9 with at least four sgRNA pairs (−52/+9, −47/−0, −47/+9, −47/+10) (Supplementary Fig. 2a) or AsCas12a in with crRNAs −2 or +11 used individually or in combination with another crRNA (Supplementary Fig. 2b). Analysis of the deletions induced by sgRNA pairs showed that SpCas9 efficiently cuts the expected DNA fragments (Supplementary Fig. 2c). Conversely, weak deletion products were detected with AsCas12a in samples where splicing was not repaired (compare Supplementary Fig. 2b and d).

To further validate the activity of SpCas9 or AsCas12a with the selected sgRNAs/crRNAs within a more physiological chromatin context, we tested the splicing correction of *CFTR* intron 19 in HEK293 cells stably transfected with the pMG3272-26A>G minigene (HEK293/3272-26A>G). Unexpectedly, all the SpCas9–sgRNA pairs failed to correct the splicing defect, suggesting inefficient cleavage at the chromosomal level (Supplementary Fig. 2e, f). Conversely, the AsCas12a-crRNA+11 generated high amounts of correct transcripts from the pMG3272-26A>G transgene (more than 60%, Fig. 1b, c and Supplementary Fig. 2g) and efficient DNA editing (67.4%, Fig. 1d).

The tracking of indels by decomposition (TIDE) analysis of the integrated minigenes^38^, following editing with AsCas12a-crRNA+ 11, revealed a heterogeneous pool of deletions (Supplementary Fig. 3a). The edited variants were cloned into the pMG3272-26A>G minigene to analyze the individual editing events and their derived splicing products (Fig. 1e). Sequence analysis of the edited sites showed a high frequency of 18 nucleotides deletions, which is consistent with the editing profile of AsCas12a (deletions bigger than four bases^20^), along with the persistence of the 3272-26A>G mutation (Fig. 1e and Supplementary Fig. 3a). Notably, the splicing analysis revealed that the frequent 18 nucleotides deletion (9/34 clones) fully restored the correct splicing (Fig. 1e and Supplementary Fig. 3b). Most of the remaining edited sites, occurring at low frequency (1/34 clones), generated a correct splicing, accompanied in few cases by an additional transcript product (Supplementary Fig. 3b). Overall, the large majority (68%) of analyzed editing events contributed to the effective restoration of normal splicing in the bulk cell population. In silico analysi*s*^39,40^ of the most frequent editing events (above 1% indel frequency) shows that the large majority of the indels decrease the strength of the cryptic splice sites activated by the 3272-26A>G CF mutation (Supplementary Fig. 3c).

In conclusion, AsCas12a in combination with a single guide RNA (crRNA+11) generates small deletions upstream of the 3272-26A>G mutation in a minigene model, producing efficient recovery of the CF splicing defect.

### Precision of the AsCas12a-based 3272-26A>G correction

The large majority of CF patients are compound heterozygous for the 3272-26A>G mutation, thus requiring a careful evaluation of the potential AsCas12a-crRNA+11 modifications within the other mutant allele, having the wild-type 3272–26 sequence.

**Fig. 2:**
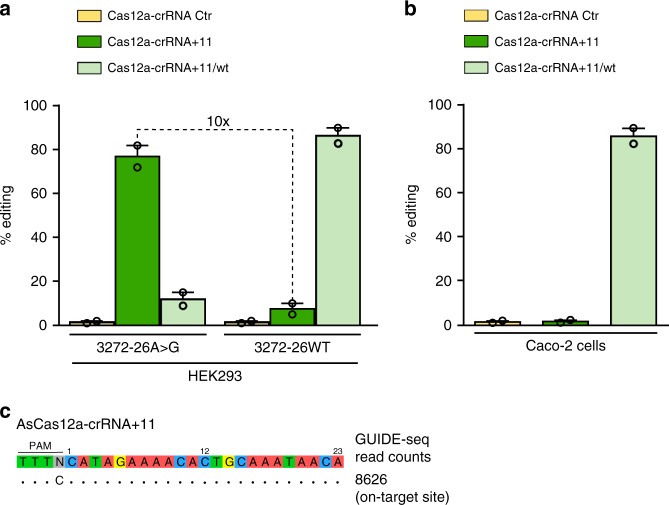
Target specificity of AsCas12a-crRNA+11. **a**, **b** Editing efficiency by TIDE analysis in HEK293/pMG3272-26WT or HEK293/pMG3272-26A>G **a** and in Caco-2 cells **b** following lentiviral transduction of AsCas12a-crRNA+11 or +11/wt, as indicated. Data are means ± SEM from *n* = 2 independent experiments. **c** GUIDE-seq analysis of crRNA+11

The cleavage properties of the AsCas12a-crRNA+11 were analyzed in stable cell lines expressing either pMG3272-26WT or pMG3272-26A>G (HEK293/3272-26WT and HEK293/3272-26A>G cells, respectively). As shown in Fig. 2a, cleavage efficiency of crRNA+11 dropped from 77%, detected in HEK293/3272-26A>G, to 7.5% in HEK293/3272-26WT, demonstrating at least 10-fold differential cleavage between the mutant and wild-type allele. Reciprocal experiments with crRNA+11/wt, targeting the *CFTR* 3272-26WT sequence showed high cleavage efficiency (86.5%) in HEK293/3272-26WT and low indels formation (12%) in HEK293/3272-26A>G (Fig. 2a), thus demonstrating high allelic discrimination by AsCas12a with the selected crRNA.

The specificity of the AsCas12a-crRNA+11 delivered by lentiviral vectors towards the wild type intron, was further confirmed in Caco-2 epithelial cells endogenously expressing the wild type *CFTR* gene. Long-term nuclease expression (10 days after transduction), which has been demonstrated to highly favor non-specific cleavages^41^, did not generate any unspecific *CFTR* editing above TIDE background levels (about 1%)^38^; whereas AsCas12a-crRNA+11/wt efficiently edited the *CFTR* gene (86.3%, Fig. 2b).

To exclude splicing alterations following potential wild-type intronic cleavages, the splicing pattern was evaluated in HEK293/3272-26WT and Caco-2 cells: no major alterations were observed following AsCas12a treatment in combination with either crRNA+ 11/wt or crRNA+11 (Supplementary Fig. 4a, b).

The specificity of the AsCas12a-crRNA+11 was also tested in terms of off-target cleavages by a genome-wide survey, GUIDE-seq^35,36^. Off-target profiling of AsCas12a-crRNA+11 genome editing in HEK293/3272-26A>G cells^18,42^ showed very high specificity, as demonstrated by exclusive editing of the 3272-26A>G *CFTR* locus, while non-specific cleavages in the second allele, or any other genomic loci, could not be detected (Fig. 2c and Supplementary Fig. 4c).

### 3272-26A>G splicing correction in primary airway cells

**Fig. 3:**
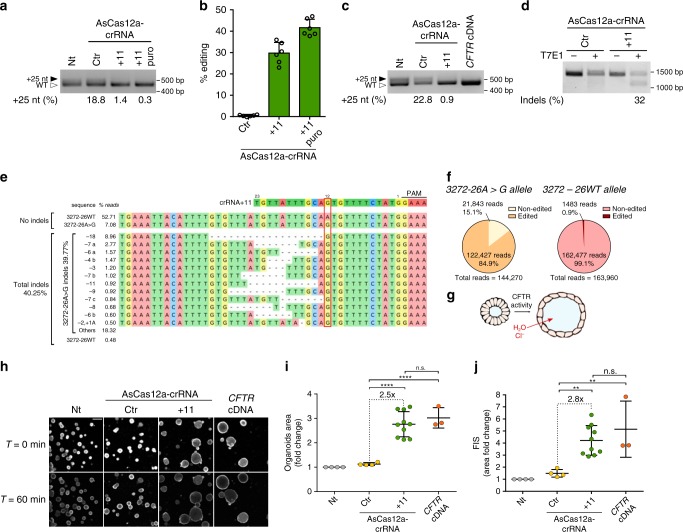
AsCas12a-crRNA+11 genome editing analysis in 3272-26A>G models. **a** Splicing pattern analysis by RT-PCR in 3272-26A>G primary airway cells following lentiviral transduction (15 days) of AsCas12a-crRNA control (Ctr) or specific for the 3272-26A>G mutation (+11). Puromycin selection was performed for 72h in +11 puro. Black-solid arrow indicates aberrant splicing, white-empty arrow indicates correct splicing. The percentages of aberrant splicing (25 nt insertion into mRNA) was measured by chromatogram decomposition analysis (Supplementary Fig. 5). **b** Percentages of indels generated in intron 20 by crRNA+11 in cells treated as in **a** measured by TIDE analysis. Data are from *n* = 2 independent experiments. **c** Splicing pattern analysis by RT-PCR in 3272-26A>G intestinal organoids following lentiviral transduction (14 days) of AsCas12a-crRNA control (Ctr) or specific for the 3272-26A>G mutation (+11) or with the *CFTR* cDNA. Black-solid arrow indicates aberrant splicing, white-empty arrow indicates correct splicing. The percentages of aberrant splicing were measured as in **a** (Supplementary Fig. 6a, b). **d** Editing efficiency in 3272-26A>G organoids measured by T7E1 assay following lentiviral transduction as in **c**. **e** Deep sequencing analysis of the *CFTR* on-target locus after AsCas12a-crRNA+11 transduction of the 3272-26A>G organoids (average from *n* = 2 independent experiments) (Supplementary Data 2). **f** Percentage of deep sequencing reads of the edited and non-edited 3272–26A>G or WT alleles from **e**. **g** Schematic representation of CFTR dependent swelling in primary intestinal organoids. **h** Representative confocal images of calcein green labeled 3272-26A>G organoids before (*T* = 0 min) and after (*T* = 60 min) forskolin-induced swelling (FIS) assay (5 µM forskolin). Scale bar = 200 µm. **i** Quantification of organoid area following lentiviral transduction of AsCas12a-crRNA Ctr, AsCas12a-crRNA+11 or with *CFTR* cDNA as indicated. Each dot represents the average organoid area analyzed for a single well (number of organoids per well: 25–300) from *n* = 4 independent experiments. **j** Fold change in organoid area before (*T* = 0 min) and after (*T* = 60 min) the forskolin-induced swelling (FIS) assay, each dot represents the average increase in organoid area analyzed per well (number of organoids per well: 25–300) from *n* = 4 independent experiments. Data are means ± SD. Statistical analysis was performed using one-way ANOVA; **P < 0.01, ****P < 0.0001, n.s. non-significant

The efficacy of the 3272-26A>G correction by AsCas12a-crRNA+11 was further validated in primary airway epithelial cells derived from a patient compound heterozygous for the 3272-26A>G splicing mutation (3272-26A>G/ΔF508). Human primary airway epithelial cells are a physiologically relevant 2-D model for CF disease modeling and preclinical testing of CF therapies^43,44^. As expected, two different transcripts were detected in these cells (Fig. 3a), whose difference in size and abundance is consistent with the cells heterozygosity for the 3272-26A>G mutation and in agreement with previous data^30^. A 13-fold correction of the aberrant 3272-26A>G splicing was obtained by lentiviral delivery of AsCas12a-crRNA+11 either with or without puromycin selection (+25nt isoform: 18.8% control sample, 1.4% crRNA+11, 0.3% crRNA+11 puro; Fig. 3a and Supplementary Fig. 5a–c).

The editing efficiency was evaluated by TIDE analysis revealing 30% of indels (up to 42% following puromycin selection, Fig. 3b and Supplementary Fig. 5d). Of note, since TIDE sequencing does not distinguish between the two *CFTR* alleles (3272-26A>G/ΔF508), the allelic discrimination of our genome editing approach was evaluated by deep sequencing, revealing 78.7% editing of the 3272-26A>G allele and complete absence of indels in the second *CFTR* allele (3272-26WT allele, Supplementary Fig. 6a, b). To further evaluate the off-target profile in primary airway epithelial cells, computationally predicted off-target sites with up to four mismatches (12 sites) were analyzed by deep sequencing. Consistent with GUIDE-seq results (Fig. 2c) no off-target cleavages were observed (Supplementary Fig. 6c).

### Splicing correction in 3272-26A>G intestinal organoids

Human organoids represent a near-physiological model for translational research^45^. Intestinal organoids from CF patients are valuable tools to evaluate CFTR channel activity and functional recovery^46–48^.

Encouraged by the splicing correction and allele specificity obtained in minigene cell models and in primary airway epithelial cells, we next evaluated the rescue potential of the CF phenotype by AsCas12a-crRNA+11 in human intestinal organoids compound heterozygous for the 3272-26A>G mutation (3272-26A>G/4218insT).

As observed in primary epithelial airway cells, the splicing pattern of *CFTR* intron 19 in the crRNA control and untreated organoids showed two transcript variants (Fig. 3c). Lentiviral delivery of AsCas12a-crRNA+11 showed nearly complete disappearance of the altered splicing product generated by the 3272-26A>G allele (+25nt), indicating efficient correction of the aberrant intron 19 splicing (Fig. 3c and Supplementary Fig. 7a, b). The amount of indels induced by AsCas12a-crRNA+11 was initially evaluated by the T7 Endonuclease I assay, resulting in approximately 30% editing of the *CFTR* locus (Fig. 3d), which is consistent with the degree of restored splicing observed in Fig. 3c.

Deep sequencing analysis revealed 40.25% indels in the *CFTR* locus (39.77% within the 3272-26A>G allele and 0.48% within the other allele, Fig. 3e), thus confirming the high efficiency of AsCas12a-crRNA+11 editing observed with the T7 Endonuclease I assay (Fig. 3d). Further sequence analysis revealed that 84.9% of the sequencing reads including the 3272-26A>G mutation contained variable length deletions, while sequencing reads corresponding to the other allele (3272-26WT) contained only 0.9% indels, thus indicating a 94-fold allelic discrimination (Fig. 3f).

In agreement with previous reports^49,50^, and despite the heterogeneity of the observed editing, the repair events in patient’s organoids were largely similar to those observed in pMG3272-26A>G model, with the 18 nucleotide deletion as the most frequent repair (compare Fig. 3e with Fig. 1e). Notably, this 18 nucleotides deletion, as well as most of the other reported indels (with a frequency above 0.5% of total DNA repair events, Fig. 3e), generated splicing correction when cloned in the pMG3272-26 model (Fig. 1e).

Lumen formation and increased organoid size of intestinal organoids (swelling) depends on the activity of the CFTR anion channel^46^ (schematized in Fig. 3g) and thus can be used to measure the restoration of CFTR function after AsCas12a-crRNA+11 genome editing. Fourteen days post AsCas12a-crRNA+11 treatment the patient’s organoids showed a 2.5-fold increased organoid area at steady-state compared to the organoids of control and untreated samples, thus indicating restored channel function following repair of the *CFTR* 3272-26A>G allele (Fig. 3h, i). Noteworthy, there was no significant difference in organoid area between treatment with AsCas12a-crRNA+11 or transduction of WT *CFTR* cDNA (Fig. 3i), further supporting the remarkable efficiency of the AsCas12a-crRNA+11 genetic editing in 3272-26A>G phenotypic reversion.

In addition to demonstrating a rescue in steady-state CFTR function (organoid swelling after treatment with AsCas12a-crRNA+11), CFTR function was also assessed by the well-established forskolin-induced swelling (FIS) assay^46^ (Fig. 3h, j). Consistent with the data in Fig. 3i, the FIS assay revealed an increase in AsCas12a-edited organoid area of 2.8-fold, which is similar to the results obtained with lentiviral delivery of WT *CFTR* cDNA (Fig. 3j and Supplementary Fig. 7c).

In light of these results, we conclude that AsCas12a-crRNA+11 modifications of the 3272-26A>G defect in patient’s organoids allows the repair of the intron 19 splicing defect, leading to full recovery of the endogenous CFTR protein function.

### Genetic correction of the *CFTR* 3849+10kbC>T splicing defect

To further evaluate the broader application of the developed AsCas12a-crRNA editing strategy, the *CFTR* 3849+10kbC>T splicing mutation was investigated. This genetic variant generates the inclusion of a cryptic exon of 84 nucleotides in the *CFTR* mature mRNA, which is translated into a truncated defective anion channel^33^.

**Fig. 4:**
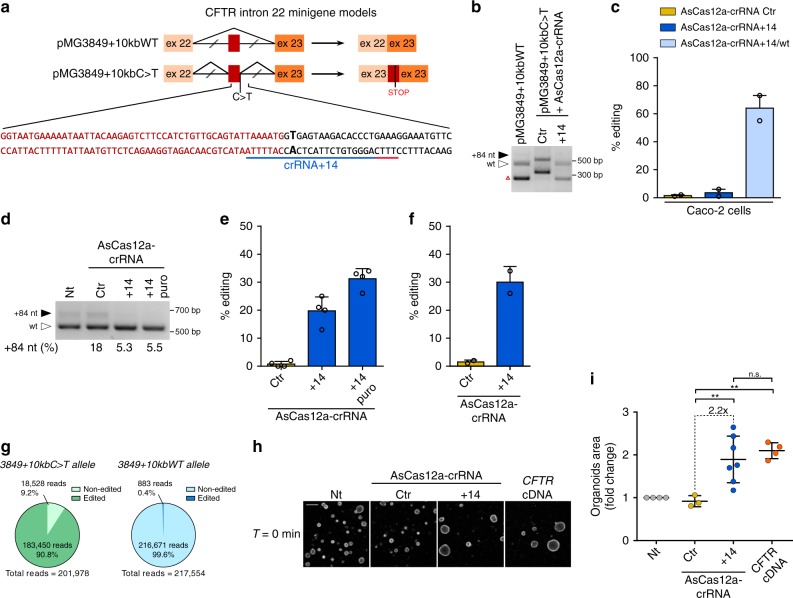
Correction of 3849+10kbC>T splicing defect by AsCas12a-crRNA+14 editing. **a** Scheme of *CFTR* wild type (pMG3849+10kbWT) and 3849+10KbC>T (pMG3849+10kbC>T) minigenes carrying exon 22, portions of intron 22 encompassing the 3849+10KbC>T, and exon 23 of the *CFTR* gene. Exons are shown as boxes and introns as lines; the expected spliced transcripts are represented on the right according to the presence or absence of the 3849+10kbC>T mutation. The lower panel shows nucleotide sequence nearby 3849+10kbC>T mutation (labeled in bold) and the AsCas12a-crRNA+14 target position (underlined, with the PAM in red). **b** Splicing pattern analyzed by RT-PCR in HEK293/pMG3849+10kbC>T cells following treatments with AsCas12a-crRNA control (Ctr) or specific for the 3272-26A>G mutation (+14). Black-solid arrow indicates aberrant splicing, white-empty arrow indicates correct splicing and red triangle indicates a minigene splicing artifact. **c** Caco-2 cells lentivirally transduced with AsCas12a-crRNA+14 or +14/wt were analyzed for editing in *CFTR* intron 22 by SYNTHEGO ICE analysis. Data are means ± SEM from *n* = 2 independent experiments. **d** Splicing pattern analyzed by RT-PCR in 3849+10kbC>T primary airway cells following lentiviral transduction (15 days) of AsCas12a-crRNA control (Ctr) or specific for the 3849+10kbC>T mutation (+14). Puromycin selection was performed for 72 h in +14 puro. The percentage of aberrant splicing (84 nt insertion into mRNA) was measured by densitometric analysis. **e** Percentages of indels in 3849+10kbC>T primary airway cells measured by TIDE analysis following lentiviral transduction as in **d**. Data are from *n* = 2 independent experiments. **f** 3849+10Kb C>T patient derived intestinal organoids were lentivirally transduced with AsCas12a-crRNA control (Ctr) or crRNA+14 and analyzed for intron 22 editing by SYNTHEGO ICE analysis. Data are from *n* = 1 experiment. **g** Percentage of deep sequencing reads of the edited and non-edited 3849+10kbC>T or WT alleles from **f**. **h** Confocal images of calcein green labeled 3849+10KbC>T organoids transduced with AsCas12a-crRNA+14 or *CFTR* cDNA. Scale bar 200 µm. **i** Quantification of organoid area; each dot represents the average area of organoids analyzed in each well (number of organoids per well: 3–30) from *n* = 1 experiment. Data are means ± SD. Statistical analysis was performed using one-way ANOVA; **P < 0.01, n.s. non-significant

We generated minigene models (pMG3849+10kbWT and pMG3849+10kbC>T) containing exon 22, part of intron 22 and exon 23 (legacy name: exon 19, intron 19 and exon 20) (schematized in Fig. 4a) that were demonstrated to mimic either wild-type or defective *CFTR* splicing (Supplementary Fig. 8a–c). The crRNA+14, targeting the 3849+10kbC>T mutation, showed a complete correction of the altered splicing in combination with AsCas12a in the minigene model (Fig. 4b). Moreover, lentiviral transduction of AsCas12a-crRNA+14 in Caco-2 cells, generated indels (3.5%) near background levels in the wild-type *CFTR* gene, while the AsCas12a-crRNA+14/wt, targeting the wild-type sequence in the same region, produced 64% *CFTR* editing, thus indicating specificity of the AsCas12a-crRNA+14 towards the mutant allele (Fig. 4c).

To further verify the AsCas12a-crRNA+14 specificity, also in terms of genome-wide off-target activity, GUIDE-seq analysis was performed in HEK293T cells, showing the complete absence of sequence reads in the *CFTR* locus or in any other off-target site; the 631 sequencing reads corresponding to spontaneous DNA breaks are indicative of the proper execution of the GUIDE-seq assay (Supplementary Fig. 4c).

The CFTR splicing pattern was then analyzed in airway epithelial cells derived from a compound heterozygous patient carrying both the 3849+10kbC>T and the ΔF508 mutations. The aberrant splicing generated by the 3849+10kbC>T mutation (+84 nt transcript) could be reversed following lentiviral delivery of the mutation specific AsCas12a-crRNA+14 (Fig. 4d), which correlated with 20% indels (30% after puromycin selection, Fig.4e). Allelic discrimination of crRNA+14 was evaluated by deep sequencing, revealing 70.7% cleavages in the 3849+10kbC>T locus and fully preserved second *CFTR* allele (3849+10kbWT allele, Supplementary Fig. 9a, b). The editing precision was further evaluated through deep-sequencing analysis of three predicted off-target sites (up to four mismatches), showing complete absence of non-specific cleavages (Supplementary Fig. 9c) as observed with the GUIDE-seq analysis in HEK293T cells (Supplementary Fig. 4c).

The genome editing strategy was verified in intestinal organoids, derived from a compound heterozygous patient carrying the 3849+10kbC>T mutation (3849+10kbC>T/ΔF508). Lentiviral delivery of AsCas12a crRNA+14 produced 30% of indels in the *CFTR* loci modifying the aberrant splicing site (Fig. 4f, g and Supplementary Fig. 10a, b), leading to the rescue of organoid swelling as strong as the one observed after *CFTR* cDNA addition (Fig. 4h, i). Sequencing analysis aimed at evaluating allelic discrimination, confirmed the extreme precision of this genome editing approach (Fig. 4g and Supplementary Fig. 9d).

Finally, we evaluated the editing efficacy of SpCas9 using the pMG3849+10kbC>T minigene. We found that, consistently with the most commonly used genome editing strategies^27,51^, SpCas9 reversed the splicing defects exclusively with sgRNA pairs which deleted the intronic region containing the mutation (Supplementary Fig. 11a, b); in contrast to AsCas12a, SpCas9 combined with an individual sgRNA targeting the mutation (+1, +5, +18) did not repair the splicing defect (Supplementary Fig. 11a).

The best identified sgRNAs pair, −95/+119, was selected among those generating the expected deletion in the *CFTR* locus and specifically repairing the aberrant splicing (Supplementary Fig. 11c–e). This SpCas9–sgRNA pair induced an increase in organoid area which was significantly lower than the increase observed in organoids after lentiviral delivery of the *CFTR* cDNA (Supplementary Fig. 11f, g), thus suggesting a lower efficacy than the one obtained with AsCas12a (Fig. 4h, i). Moreover, in contrast to the allele specificity of the single AsCas12-crRNA+14, the SpCas9–sgRNA pairs, which are necessary for functional correction, produced deletions also in the non-targeted second allele (ΔF508). Therefore, the final genome editing efficacy should be considered diluted over the two alleles. In addition, although our sgRNA pool was designed in silico to minimize the probability of SpCas9 off-target activity^52^, the GUIDE-seq assay for sgRNA+119 revealed 11 off-target sites throughout the genome (Supplementary Figs. 11h and 4d).

In conclusion, similarly to the splicing repair of the 3272-26A>G variant, the correction of the *CFTR* 3849+10kbC>T splicing defect was efficiently and precisely obtained by using AsCas12a combined with a single allele specific crRNA in CF patient-derived organoids. This strategy was proven superior to the conventional SpCas9-induced genetic deletion obtained using sgRNA pairs.

## Discussion

The most advanced strategies so far developed for CF gene therapy are based on the delivery, preferably in the lungs, of a copy of the *CFTR* cDNA to compensate patients’ defective *CFTR* gene. The main limitation of this gene therapy strategy is the low and non-permanent CFTR expression obtained in the affected tissues^7,53^. CFTR expression below therapeutic benefit is mainly due to loss of the trans-gene during the rapid turnover of pulmonary epithelial cells and inefficient lung transduction; conditions that are further worsened by disease symptoms in CF patients. Viral and non-viral vectors have proven valid to deliver the *CFTR* cDNA in cell and animal models of CF^8,10^. Among these are the adenoviral and adeno-associated-viral (AAV) delivery systems which, however, are either associated with immune responses or necessitate multiple treatments due to their transient cellular persistence^54,55^. Lentiviral vectors recently reached the clinic for the treatment of a severe combined immunodeficiency^56^. This delivery system with improved genome safety features over the original retroviral vectors, offer significant advantages in terms of deliverability in both dividing and non-dividing cells. In the CF clinical field recent pre-clinical studies provided encouraging results on lentiviral-mediated *CFTR* delivery which are leading to the preparation of a first-in-man lentivirus trial in patients with CF^11,57^.

Latest advances in genome editing, mainly represented by CRISPR-nucleases, offer the unprecedented opportunity to efficiently repair genetic defects within the endogenous *CFTR* locus to permanently restore the physiological expression of the gene. Furthermore, genetic correction does not require continuous expression of the nuclease thereby bypassing the need for a stable transgene expression^58^. Several methods have been described to limit continuous expression of lentiviral transgenes as natural promoter silencing and self-limiting CRISPR-Cas genetic circuits^41,59,60^.

The CRISPR-nuclease SpCas9 was harnessed to correct the ΔF508 mutation by homology-directed repair (HDR)^22^. Nevertheless, the low efficiency of HDR in human cells and the requirement of the additional delivery of a DNA donor template pose several hurdles for a future clinical translation. However, the delivery of CRISPR-SpCas9 with multiple sgRNAs was shown to correct *CFTR* intronic splicing mutation in cellular minigene models by inducing deletions, although quite large in size (around 150–200 bp), of the intronic mutation and surrounding sequences^27^.

In our study, we developed a genome editing strategy using one of the most precise programmable nucleases^18^, AsCas12a, that permanently corrects *CFTR* splicing defects of at least two relevant splicing mutations (3272-26A>G and 3849+10kbC>T) in combination with a single crRNA. This approach is based on small deletions (about 4–26 nt) within intronic sequences which remove essential splicing regulatory elements forming aberrant 3′ or 5′ cryptic splice sites. Indeed, our sequencing data indicate that the AsCas12a strategy likely inactivates the cryptic 3′ splice site (poly-pyrimidine-T-rich-tract and last essential 3′-intronic nucleotides) activated by the *CFTR* 3272-26A>G mutation and the 5′ cryptic donor splice site in intron 22 activated by the 3849+10kbC>T. This was confirmed by the in silico analysis of the splicing signals.

As opposed to AsCas12a, SpCas9 splicing repair required multiple sgRNAs thus strictly depending on efficient concomitant cleavages of the target sites and efficient co-delivery by the delivery vector.

The high-fidelity AsCas12a genetic repair^18^ applied in this study to *CFTR* splicing defects, resulted in an allele specific and genome-wide off-target free editing. Allelic discrimination was obtained by designing the crRNA target sequence on the mutations (3272-26A>G and 3849+10kbC>T), thus providing the relevant advantage of minimizing the potential risk of on-target chromosomal translocations^61^.

We demonstrated that our AsCas12a-based gene correction strategy efficiently corrects the splicing pattern in human primary airway epithelial cells and rescued endogenous CFTR function in patient derived intestinal organoids, which are recognized as a highly valuable preclinical model to predict ex vivo therapeutic efficacy in CF patients^47^, providing an important milestone for future clinical trials. Even though CFTR modulators (i.e. potentiator VX-770, Kalydeco, Vertex) are used in clinic also for CF patients carrying the splicing mutations of this study, these drugs are associated with side effects and strongly depend on the residual correct splicing of mutant CFTR, which is extremely reduced and variable among patients^30–33^. This explains the urgent need for a permanent correction of physiologic levels of CFTR, potentially reachable with the genome editing approach described in our study.

In conclusion, this work sets a robust proof-of-concept to treat two different deep intronic mutations by genome editing using the AsCas12a-crRNA nuclease system, which can be broadened to other splicing defects in CF and even to other genetic diseases caused by splicing alterations.

## Methods

### Plasmids

Wild type and mutated minigenes for 3272-26A>G and 3849+10kbC>T mutations were cloned into previously published pcDNA3 and pCI plasmid, respectively^62,63^. Wild type minigenes (pMG3272-26WT and pMG3849+10kbWT) were obtained by PCR amplification and cloning of target regions from *CFTR* gene of HEK293T cell. Primers used are listed in Supplementary Data 4. The pMG3272-26WT plasmid includes the last 23 bases of exon 18, full length exons 19 and 20, and intron 19. The addition of the last part of exon 18 was performed by semi-nested PCR: the first PCR was done with oligo TM exon 18 exon 19 hCFTR fw and oligo exon 20 hCFTR rev; the second PCR was done with the forward primer oligo KpnI-AgeI exon 18–19 hCFTR fw and the same reverse oligo. The pMG3849+10kbWT plasmid contains full length exons 22 and 23, and portions of intron 22 as previously described^35^. Mutated minigenes (pMG3272-26A>G and pMG3849+10kbC>T) were obtained by site-directed mutagenesis of pMG3272-26WT and pMG3849+10kbWT, for both mutations. Plasmid sequences are described in Supplementary Data 5. Guide RNAs were cloned into pY108 lentiAsCas12a (Addgene Plasmid 84739) or lentiCRISPR v1 (Addgene Plasmid 49535) using BsmBI restriction sites as previously described^64^. These plasmids allow simultaneous delivery of the RNA-guided nuclease and the sgRNA to target cells and contain puromycin as selection marker.

### Cell lines

Human colorectal adenocarcinoma cells (Caco-2), HEK293T cells and HEK293 cells stably expressing pMG3272-26WT (HEK293/pMG3272-26WT) or 3272-26A>G cells (HEK293/pMG3272-26A>G) were cultured in Dulbecco's modified Eagle's medium (DMEM; Life Technologies) supplemented with 10% fetal bovine serum (FBS; Life Technologies), 10 U/ml antibiotics (PenStrep, Life Technologies) and 2 mM l-glutamine at 37 °C in a 5% CO_2_ humidified atmosphere. Puromycin selection was performed using 10 μg/ml for Caco-2 cells, and 2 μg/ml for HEK293T or HEK293 cells. HEK293T, HEK293, and Caco-2 cells were obtained from American Type Culture Collection (ATCC; www.atcc.org). Stable minigene cell lines (HEK293/3272–26A>G and HEK293/3272-26WT) were produced by transfection of Bgl-II linearized minigene plasmids (pMG3272-26WT or pMG3272-26A>G) in HEK293 cells. Cells were selected with 500 µg/ml of G418, 48 h after transfection. Single cell clones were isolated and characterized for the expression of the minigene construct.

### Transfection and lentiviral transduction of cell lines

Transfection experiments were performed in HEK293T cells seeded (150,000 cells/well) in a 24-well plate and transfected using polyethylenimine (PEI) with 100 ng of minigene plasmids and 700 ng of plasmid encoding for nuclease and sgRNA/crRNA (pY108 lentiAsCas12a or lentiCRISPR v1). After 16 h incubation cell medium was changed, and samples were collected at 3 days from transfection.

Lentiviral particles were produced in HEK293T cells at 80% confluency in 10 cm plates. 10 µg of transfer vector (pY108 lentiAsCas12a or lentiCRISPR v1) plasmid, 3.5 µg of VSV-G and 6.5 µg of Δ8.91 packaging plasmid were transfected using PEI. After over-night incubation the medium was replaced with complete DMEM. The viral supernatant was collected after 48 h and filtered in 0.45 μm PES filter. Lentiviral particles were concentrated and purified with 20% sucrose cushion by ultracentrifugation for 2 h at 4 °C and 150,000 × *g*. Pellets were resuspended in appropriate volume of OptiMEM. Aliquots were stored at −80 °C. Vector titres were measured as reverse transcriptase units (RTU) by SG-PERT method^65^.

For transduction experiments HEK293/pMG3272-26WT or HEK293/pMG3272-26A>G and Caco-2 cells were seeded (300,000 cells/well) in a 12-well plate and the day after were transduced with three RTU of lentiviral vectors. 48 h later, cells were selected with puromycin (2 μg/ml for HEK293 or 10 μg/ml for Caco-2 cells) and collected 10 days from transduction.

### Transcripts analysis

RNA was extracted using TRIzol™ Reagent (Invitrogen) and resuspended in DEPC-ddH2O. cDNA was obtained starting from 500 ng of RNA using RevertAid Reverse Transcriptase (Thermo Scientific), according to manufacturer’s protocol. Target regions were amplified by PCR with Phusion High Fidelity DNA Polymerase (Thermo Fisher). Oligonucleotides are listed in Supplementary Data 4. Uncropped and unprocessed scans are available in the Source Data files.

### Detection of nuclease-induced genomic mutations

Genomic DNA was extracted using QuickExtract DNA extraction solution (Epicentre) and the target locus amplified by PCR using Phusion High Fidelity DNA Polymerase (Thermo Fisher). Oligos are listed in Supplementary Data 4. To evaluate indels resulting from cleavage of one gRNA, purified PCR products were sequenced and analyzed using the TIDE or the SYNTHEGO ICE software^38,66^. In some experiments DNA editing was measured also by T7 Endonuclease 1 (T7E1) assay (New England BioLabs) following manufacturer’s instructions and as previously described^41^.

### Primary airway epithelial cell culture and transduction

Primary airway (bronchial) epithelial cells were derived from CF patients compound heterozygous for 3272-26A>G splicing mutation (3272-26A>G/ΔF508, *n* = 1) and for 3849+10Kb C>T splicing mutation (3849+10Kb C>T/ΔF508, *n* = 1) (kindly provided by the Primary Cell Culture Service of the Italian Cystic Fibrosis Research Foundation). The Ethics Committee of the Istituto Giannina Gaslini approved this study and informed consent was obtained from all participating CF subjects. Cells were cultured in LHC9/RPMI 1640 (1:1) without serum^67^ and the day before the transduction, 50,000 cells at passage 3, were seeded into a 24-well plate previously treated with collagen. Transduction was performed with two RTU of lentiviral vectors. Puromycin selection, where indicated, was performed with 2 μg/ml, 48 h post transduction for 72 h. Cells were collected for analysis after 15 days.

### Human intestinal organoids culture and transduction

Human intestinal organoids of CF subjects compound heterozygous for 3272-26A>G splicing mutation (3272-26A>G/4218insT, *n* = 1, CF-86) and for 3849+10Kb C>T mutation (3849+10Kb C>T/ΔF508, *n* = 1, CF-110) were derived from fresh rectum suction biopsies and cultured as previously described^46^. The Ethics Committee of the University Hospital Leuven approved this study and informed consent was obtained from all participating CF subjects. Organoids cultures, at passages 10–15, were trypsinized to single cell using trypsin 0.25% EDTA (Gibco), 30,000–40,000 single cells were resuspended with 25 µl of lentiviral vector (0.25–1 RTU) and incubated for 10 min at 37 °C^8^. The same amount of Matrigel (Corning) was added and the mix plated in a 96-well plate. After polymerisation of the Matrigel drops (37 °C for 7 min), they were covered with 100 µl of complete organoid medium^46^ containing 10 µM of Rock inhibitor (Y-27632 2HCl, Sigma Aldrich, ref. Y0503) for the first 3 days to ensure optimal outgrowth of single stem cells^48^. Medium was replaced every 2–3 days until the day of organoid analysis.

### Analysis of CFTR activity in intestinal organoids

Fourteen days after viral vector transduction, organoids were incubated for 30 min with 0.5 µM calcein-green (Invitrogen, ref. C3-100MP) and analyzed by confocal live cell microscopy with a ×5 objective (LSM800, Zeiss, with Zen Blue software, version 2.3). Steady-state organoids area was determined by calculating the absolute area (*xy* plane, µm^2^) of each organoid using ImageJ software through the Analyze Particle algorithm. Defective particles with an area <1500 or 3000 µm for 3272-26A>G or 3849+10Kb C>T, respectively, were excluded from the analysis. Data were averaged for each different experiment and plotted in a box plot representing means ± SD.

The FIS assay was performed by stimulating organoids with 5 µM of forskolin and analyzed by confocal live cell microscopy at 37 **°**C for 60 min (one image every 10 min). The organoid area (*xy* plane) at different time points was calculated using ImageJ, as described above.

### GUIDE-seq

GUIDE-seq experiments were performed as previously described^42^. Briefly, 2 × 10^5^ HEK293T cells were transfected using Lipofectamine 3000 transfection reagent (Invitrogen) with 1 µg of lenti Cas12a plasmid (pY108) and 10 pmol of dsODNs^42^. The day after transfection cells were detached and selected with 2 µg/ml puromycin. Four days after transfection cells were collected and genomic DNA extracted using DNeasy Blood and Tissue kit (Qiagen) following manufacturer’s instructions. Using Bioruptor Pico sonicatin device (Diagenode) genomic DNA was sheared to an average length of 500 bp. Library preparations were performed with the original adapters and primers according to previous work. Libraries were quantified with the Qubit dsDNA High Sensitivity Assay kit (Invitrogen) and sequenced with the MiSeq sequencing system (Illumina) using an Illumina Miseq Reagent kit V2-300 cycles (2 × 150 bp paired-end). Raw sequencing data (FASTQ files) were analyzed using the GUIDE-seq computational pipeline^42^. After demultiplexing, putative PCR duplicates were consolidated into single reads. Consolidated reads were mapped to the human reference genome GrCh37 using BWA-MEM; reads with mapping quality lower than 50 were filtered out. Upon the identification of the genomic regions integrating double-stranded oligodeoxynucleotide (dsODNs) in aligned data, off-target sites were retained if at most seven mismatches against the target were present and if absent in the background controls. Visualization of aligned off-target sites is available as a color-coded sequence grid^23,26^. GUIDE-seq data are listed in Supplementary Data 3.

### Targeted deep sequencing

The loci of interest were amplified using Phusion high-fidelity polymerase (Thermo Scientific) from genomic DNA extracted from human intestinal organoids (3272-26A>G/4218insT) 14 days after transduction with lentiAsCas12a-crRNA +11, +14 or Ctr, either in organoids (on-target) or airway cells (on and off-target). Amplicons were indexed by PCR using Nextera indexes (Illumina), quantified with the Qubit dsDNA High Sensitivity Assay kit (Invitrogen), pooled in near-equimolar concentrations and sequenced on an Illumina Miseq system using an Illumina Miseq Reagent kit V3−150 cycles (150 bp single read). Primers used to generate the amplicons are reported in Supplementary Data 4. Raw sequencing data (FASTQ files) were analyzed using CRISPResso online tool^68^, by setting Windows size = 3, Minimum average read quality (phred33 scale) = 30 and minimum single bp quality (phred33 scale) = 10 (Supplementary Data 2).

### In silico off-target analysis

Off-target for crRNA +11 and +14 were analyzed by Cas-OFFinder online algorithm, by selecting: AsCpf1 from *Acidaminococcus* or LbCpf1 from *Lachnospiraceae* 5'-TTTV-3', mismatch number≤4, DNA bulge size = 0 and as a target genome the *Homo sapiens* (GRCh38/hg38)—Human.

### In silico splicing prediction

For wild-type, mutated and edited CFTR gene sequences, a region of 400 bp spanning either the 3272-26A>G or 3849+10Kb C>T locus was analyzed by HSF and MaxEnt prediction algorithms^39,40^ available at the Human Splicing Finder website (www.umd.be/HSF3/). Splice sites score were normalized to the score of the 3272-26A>G, or 3849+10Kb C>T splice site (Supplementary Data 6).

### Statistical analyses

Statistical analyses were performed by GraphPad Prism version 6. For organoids experiments ordinary one-way analysis of variance (ANOVA) was performed. For the in silico prediction analyses Shapiro–Wilk test was used to verify the distribution of the data. Significance of the data was calculated by two-tailed Wilcoxon signed-rank test. Differences were considered statistically different at *P* < 0.05.

### Reporting summary

Further information on research design is available in the Nature Research Reporting Summary linked to this article.

## Supplementary information

Supplementary Information

Data 1

Data 2

Data 3

Data 4

Data 5

Data 6

Reporting Summary

Description of Additional Supplementary Files

Source data

## Data Availability

GUIDE-seq and targeted deep-sequencing data have been deposited at BioProject (https://www.ncbi.nlm.nih.gov/bioproject/) under the accession number PRJNA551109. The source data underlying Figs. 1b–e, 2a, b, 3a–d, j, k, 4b–f, i and Supplementary Figs. 1a, 2e-f, 3a, b, 4a, b, 5a–c, 7b, c, 8a, 11a–e, g are provided as a Source Data file. All other relevant data are available from the authors upon reasonable request.
