# Author Correction: Allele specific repair of splicing mutations in cystic fibrosis through AsCas12a genome editing

**DOI:** 10.1038/s41467-020-19351-2

**Published:** 2020-10-22

**Authors:** Giulia Maule, Antonio Casini, Claudia Montagna, Anabela S. Ramalho, Kris De Boeck, Zeger Debyser, Marianne S. Carlon, Gianluca Petris, Anna Cereseto

**Affiliations:** 1 Centre for Integrative Biology (CIBIO), University of Trento, Via Sommarive 9, 38123 Trento, Italy; 2 Department of Development and Regeneration, CF Centre, Woman and Child, KU Leuven, Herestraat 49, Leuven, 3000 Belgium; 3 Pediatric Pulmonology, Department of Pediatrics, University Hospital Leuven, Herestraat 49, Leuven, 3000 Belgium; 4 Laboratory for Molecular Virology and Drug Discovery, Department of Pharmaceutical and Pharmacological Sciences, KU Leuven, Herestraat 49, Leuven, 3000 Belgium; 5 Present Address: Medical Research Council Laboratory of Molecular Biology, Cambridge Biomedical Campus, Francis Crick Avenue, Cambridge, CB2 0QH UK

**Keywords:** Cystic fibrosis, CRISPR-Cas9 genome editing

Correction to: *Nature Communications* 10.1038/s41467-019-11454-9, online 7 August 2019.

The original version of this articles omitted a reference to previous work on the early use of lentiviral vectors in the clinic, reported in Biffi et al. *Science* **341**, 10.1126/science.1233158 (2013); Epub 11 Jul 2013) . This has now been added as reference number 57. In the previous version of the article, the article text stated: ‘Lentiviral vectors recently reached the clinic for the treatment of a severe combined immunodeficiency^56^’. This text has been corrected to state: ‘Lentiviral vectors recently reached the clinic for the treatment of different types of genetic diseases^56,57^’.

This has been corrected in both the PDF and HTML versions of the Article.

