# Supplementary material for: Allele specific repair of splicing mutations in cystic fibrosis through AsCas12a genome editing
Source: Nat Commun. 2019 Aug 7;10:3556. doi: 10.1038/s41467-019-11454-9 (PMC6685978; doi:10.1038/s41467-019-11454-9)
Supplement: Supplementary file 1 — Supplementary Information [file 41467_2019_11454_MOESM1_ESM.pdf]

Supplementary Information for

Allele specific repair of splicing mutations in cystic fibrosis through AsCas12a genome editing.

Maule et al.

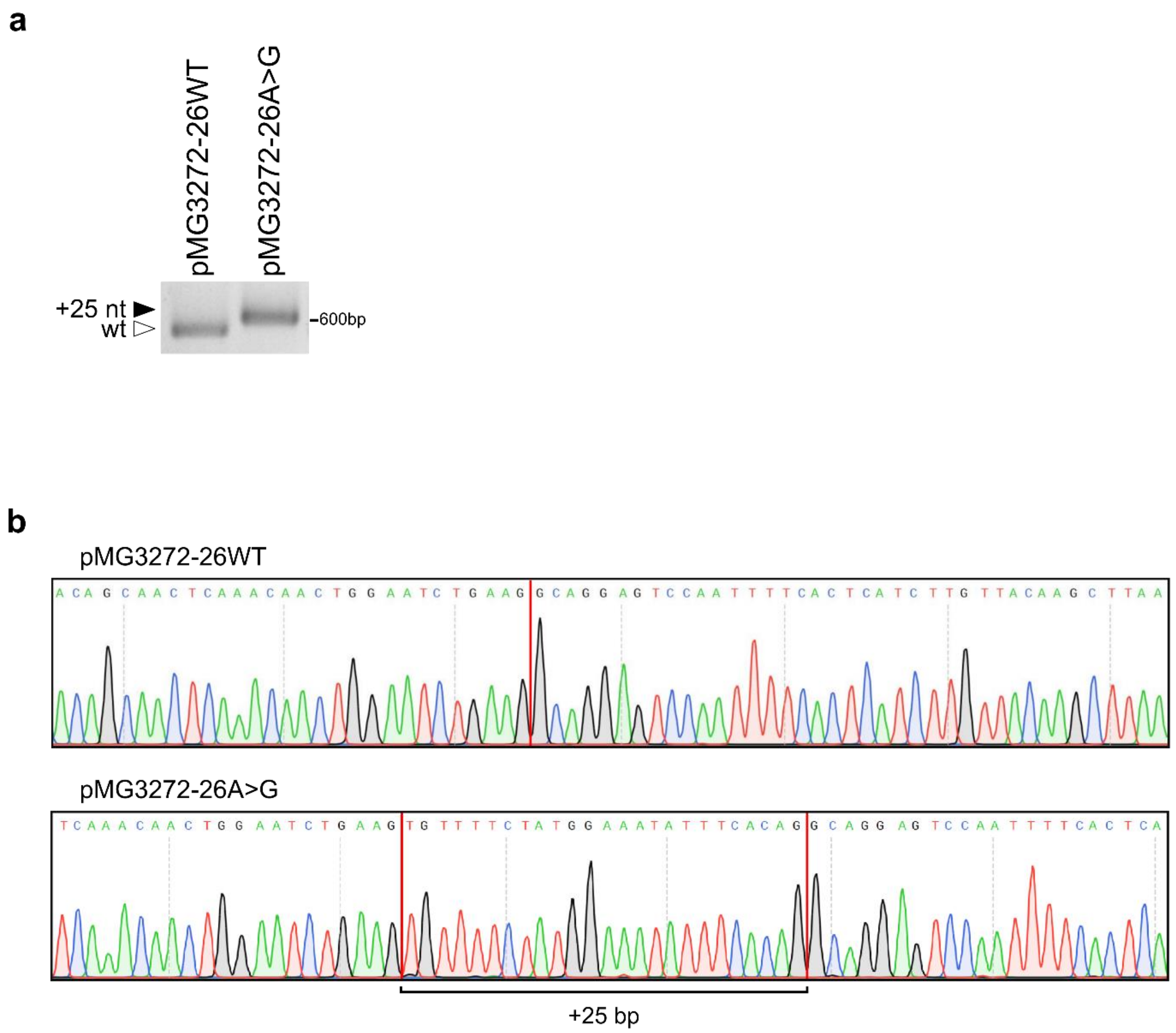

Supplementary Figure 1. Validation of intron 19 splicing in pMG3272-26WT and pMG3272-26A>G *CFTR* minigene models.

a, Splicing pattern of *CFTR* wild type (pMG3272-26WT) and mutated (pMG3272-26A>G) minigene models, transfected in HEK293T, by agarose gel electrophoresis analysis of RT-PCR products. Black-solid arrow indicates aberrant splicing, white-empty arrow indicates correct splicing. **b**, Sanger sequencing chromatogram of minigene splicing products from (**a**). Red lines represent the boundary between exons.

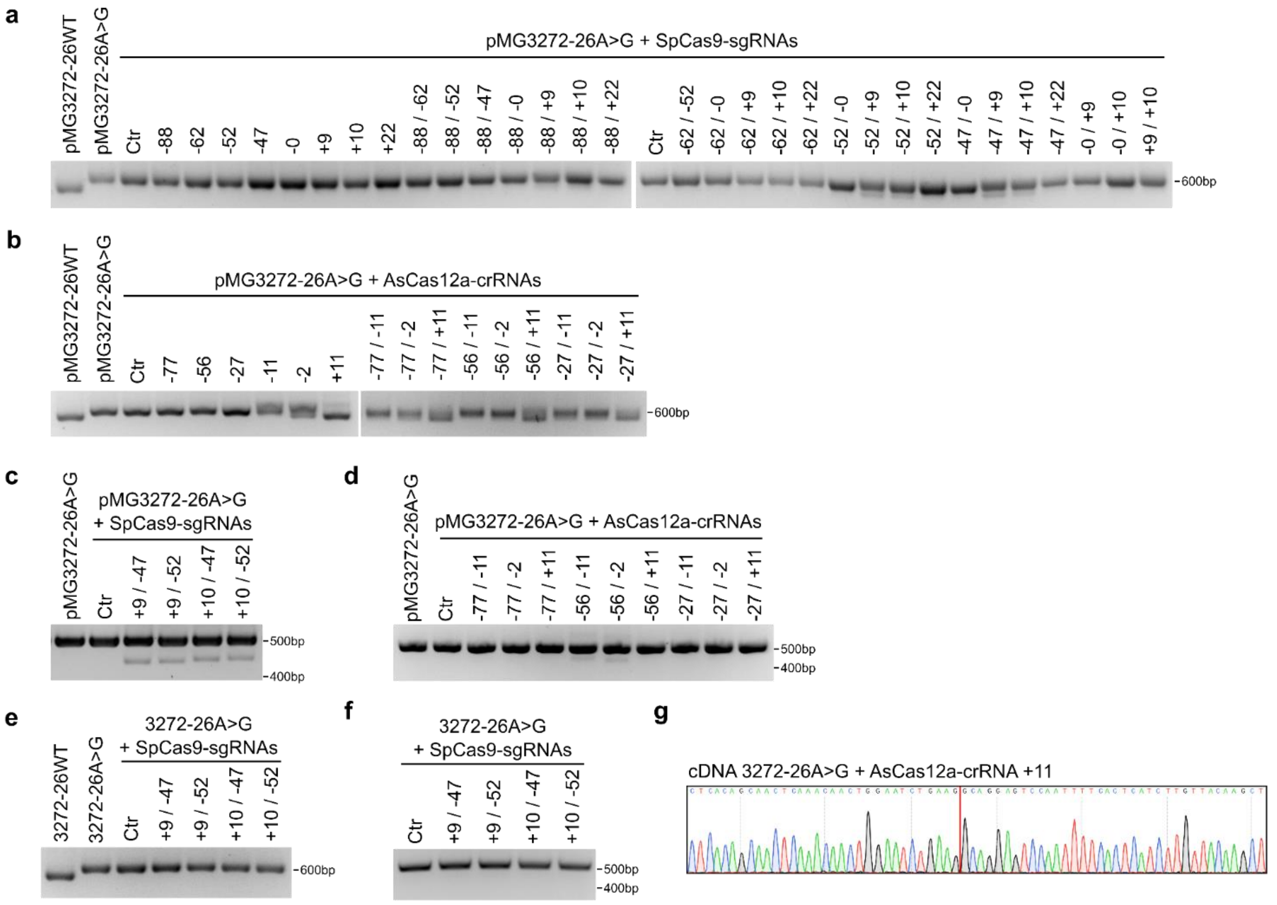

Supplementary Figure 2. SpCas9 and AsCas12a sgRNA/crRNA functional screening for splicing correction of 3272-26A>G minigene.

a,**b**, SpCas9-sgRNAs (**a**) and AsCas12a-crRNAs (**b**) screening based on the ability to restore the correct splicing pattern of *CFTR* 3272-26A>G minigene. Nucleases and sgRNAs single or in pair were transfected in HEK293T with pMG3272-26A>G, RT-PCR products were analyzed by agarose gel electrophoresis. pMG3272-26WT was used as a reference for correct intron 19 splicing. **c**,**d**, Agarose gel electrophoretic analysis of targeted deletions in 3272-26A>G minigene after cleavage by SpCas9 (**c**) and AsCas12a (**d**) sgRNA-pairs from (**a**) and (**b**), respectively, measured by PCR. The bigger band represents non-edited minigene sequences, the smaller band is the expected deletion product. **e**,**f**, Agarose gel electrophoresis of RT-PCR products (**e**) and PCR of targeted deletion (**f**) for SpCas9-sgRNA pairs selected from (**b**) in HEK293 having stable genomic integration of 3272-26A>G minigene (HEK293/pMG3272-26A>G cells). **g**, Sanger sequencing chromatogram of correct

intron 19 splicing from 3272-26A>G integrated minigene after AsCas12a-crRNA+11 editing from Fig.1b. Red line represents the boundary between exons 19-20.

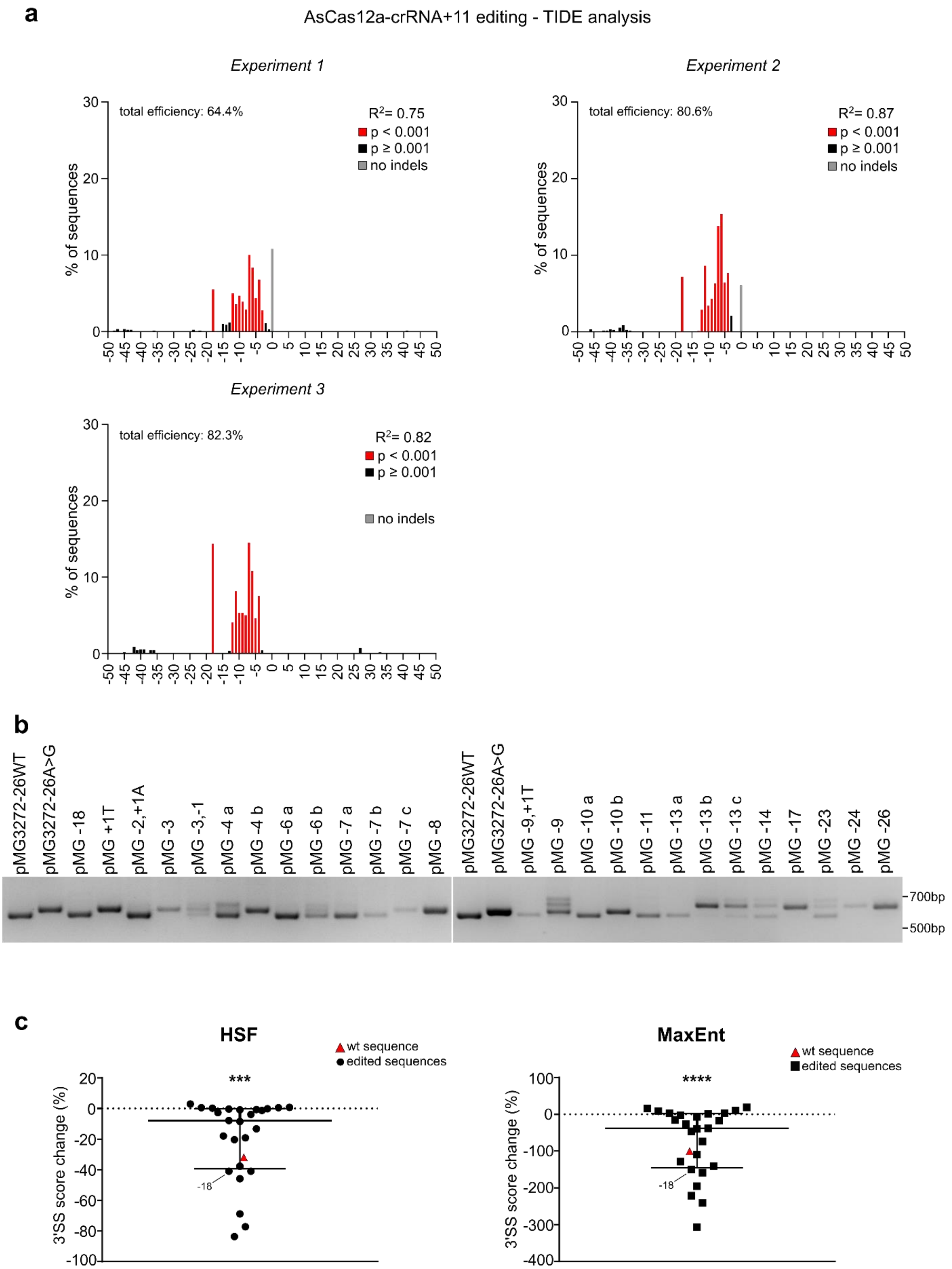
a
AsCas12a-crRNA+11 editing - TIDE analysis
Experiment 1
total efficiency: 64.4%
R²= 0.75
p < 0.001
p ≥ 0.001
no indels
% of sequences
Experiment 2
total efficiency: 80.6%
R²= 0.87
Experiment 3
total efficiency: 82.3%
R²= 0.82
b
pMG3272-26WT
pMG3272-26A>G
pMG -18
pMG +1T
pMG -2,+1A
pMG -3
pMG -3,-1
pMG -4 a
pMG -4 b
pMG -6 a
pMG -6 b
pMG -7 a
pMG -7 b
pMG -7 c
pMG -8
pMG -9,+1T
pMG -9
pMG -10 a
pMG -10 b
pMG -11
pMG -13 a
pMG -13 b
pMG -13 c
pMG -14
pMG -17
pMG -23
pMG -24
pMG -26
700bp
500bp
c
HSF
wt sequence
edited sequences
***
3'SS score change (%)
-18
MaxEnt
****

Supplementary Figure 3. Repair pattern after AsCas12a-crRNA+11 cleavage and in silico splicing score prediction.

a, Indels spectrum by TIDE analysis from HEK293/pMG3272-26A>G cells after AsCas12a-crRNA+11 editing from n=3 independent experiments. **b**, Splicing pattern of edited sites cloned into the minigene plasmid. Minigenes were transfected in HEK293T cells and RT-PCR products were analyzed by agarose gel electrophoresis. **c**, Prediction of the score change of AsCas12a-crRNA+14 modification on the mutated 3'splice site (ss) by bioinformatics tools (HSF and MaxEnt prediction algorithms, see Methods section). Data are median with interquartile range. Statistical analysis was performed using two-tailed Wilcoxon signed-rank test; ***$P<0.001$, ****$P<0.0001$. Score of wild type sequence was given only as reference in the graph.

a **b**

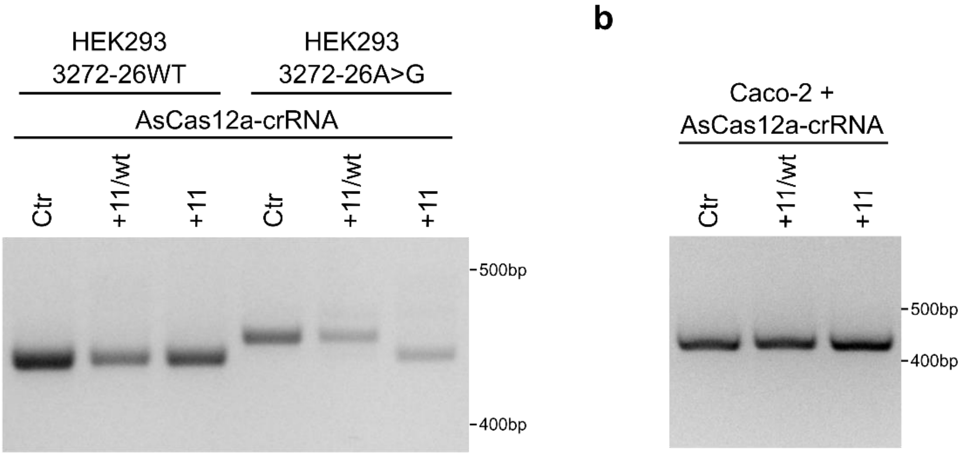

c

AsCas12a \ *TARGET*	***wt CFTR locus****	***off-targets****	***background DSBs****
crRNA +11	0	0	814
crRNA +14	0	0	631

** GUIDE-seq reads counts*

d

SpCas9 \ *TARGET*	***wt CFTR locus****	***off-target****	***background DSBs****
sgRNA +119	9669	679	3638
sgRNA -95	1729	0	4498

** GUIDE-seq reads counts*

Supplementary Figure 4. Specificity of AsCas12a-crRNA+11 or crRNA+11/wt DNA editing: unchanged wild-type CFTR splicing and GUIDE-seq analyses.

a,**b**, RT-PCR products analysis after AsCas12a-crRNA+11 or +11/wt editing in HEK293/pMG3272-26WT or A>G minigene (**a**) and in Caco-2 cells having WT *CFTR* sequence (**b**). Cells were transduced with lentiviral vector carrying AsCas12a-crRNA+11 or +11/wt and selected with puromycin for 10 days. Images are representative of two independent experiments. **c**, **d**, GUIDE-seq analysis for AsCpf1-crRNA (**c**) and SpCas9-sgRNA (**d**). The double strand breaks (DSBs) are spontaneous cleavages grouped by the analysis as spurious cleavages (not resulting from AsCas12a or SpCas9 cleavages) that are nevertheless indicative of properly executed GUIDE-seq assay.

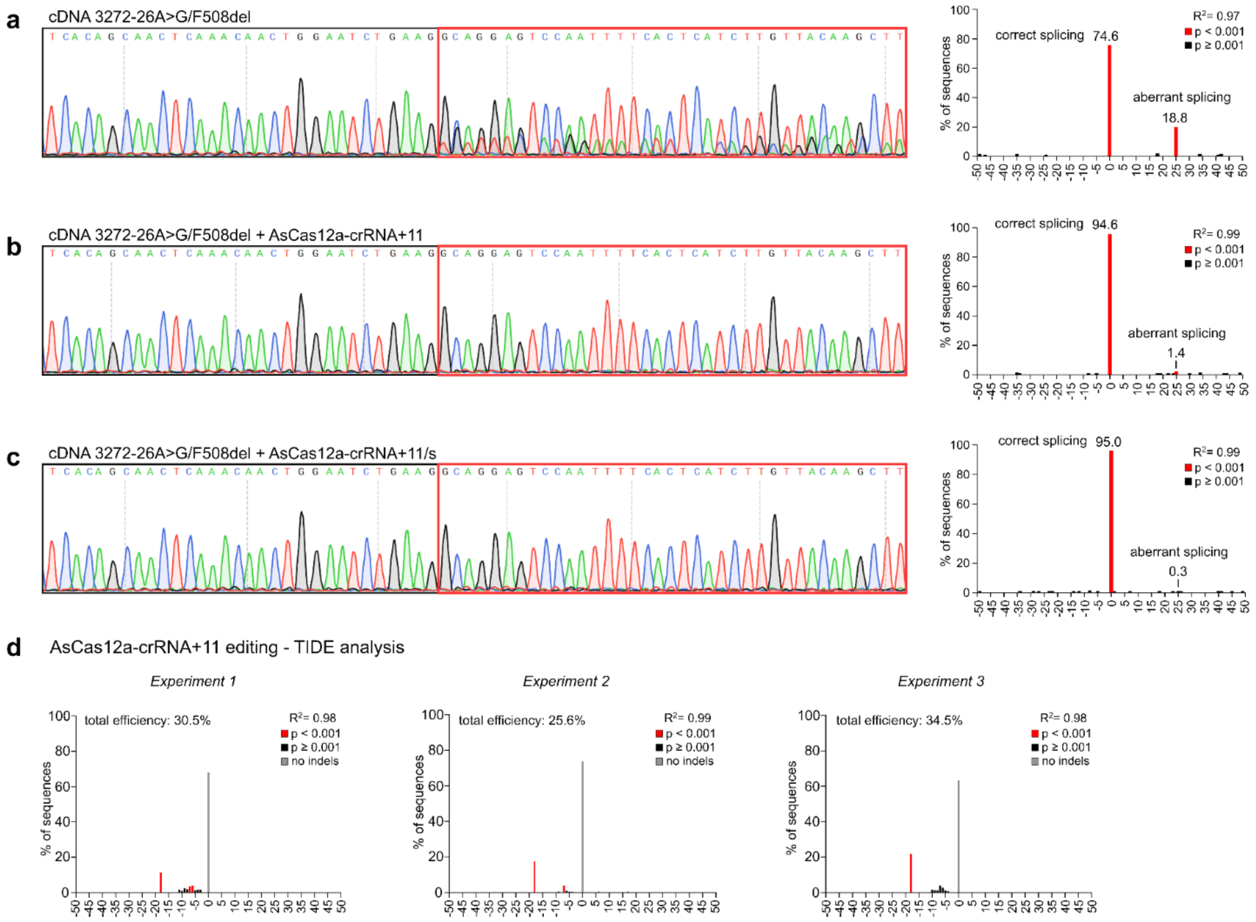

Supplementary Figure 5. Characterization of CFTR splicing and editing pattern in primary airway cells before and after AsCas12a-crRNA+11 editing.

a, **b**, **c**, Left panels, chromatograms of RT-PCR products from Ctr (**a**), AsCas12a-crRNA+11 (**b**), AsCas12a-crRNA+11-puro (**c**) samples of Fig. 3a, showing two different transcripts of 3272-26A>G/ΔF508 primary airway cells. Red box indicates chromatogram area after exon19-exon20 junction. Right panels, chromatograms deconvolution analysis was used to evaluate the amount of mutated splicing (inclusion of +25 nt from intron 19) before (**a**) and after (**b**,**c**) AsCas12a-crRNA+11 cleavage. **d**, Indels spectrum by TIDE analysis from 3272-26A>G/ΔF508 primary airway cells after AsCas12a-crRNA+11 editing from n=3 independent experiments.

a

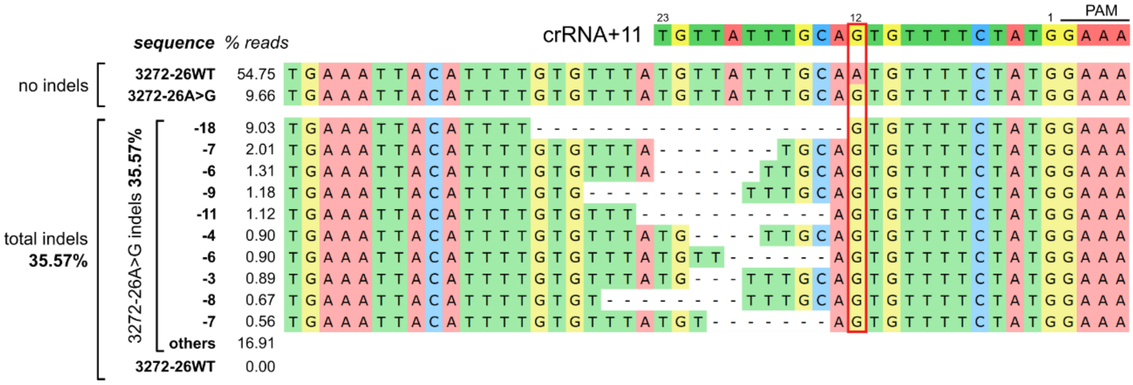

b

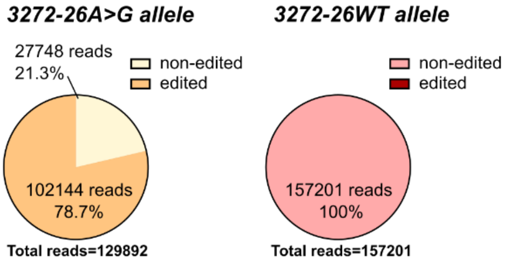

c

**AsCas12a-crRNA+11**		Chromosome	Position	Mismatches	% editing crRNA+11	total reads crRNA+11	% editing crRNA CTR	total reads crRNA CTR
3272-26A>G*	TTTCCATAGAAAACACTGCAAATAACA	chr7	117611543	0	78.7	129892	0.9	238374
3272-26WT	TTTCCATAGAAAACAtTGCAAATAACA	chr7	117611543	1	0.0	157201	0.9	241291
OFF 1	TTTCCATAGAtAAtACTGCAcATAACt	chr8	50202652	4	0.5	464287	0.4	462812
OFF 2	TTTCCAgAGAAAACACTaCAAATAAtg	chr3	176656937	4	0.4	332209	0.3	331609
OFF 3	TTTGCATAaAAAAgtCTaCAAATAACA	chr7	138131106	4	0.5	579560	0.4	498759
OFF 4	TTTACtTAGAAAAgAaTGaAAATAACA	chr4	132046364	4	0.7	713622	0.7	625136
OFF 5	TTTGCATAGAAAACACTtCcAcaAACA	chr5	25017613	4	0.7	592811	0.6	568162
OFF 6	TTTACATgGAAAAgACTGaAAATAgCA	chr5	160120802	4	0.7	434289	0.6	413415
OFF 7	TTTGCATAGAgAAtACTGCAAAaAACc	chr1	143617739	4	9.2	729449	10.1	487345
OFF 8	TTTAtATAtAAAAaACTtCAAATAACA	chr2	52000516	4	0.5	259045	0.5	581289
OFF 9	TTTACATAGAAAAaACTaaAAATgACA	chr15	70700393	4	0.7	45008	0.6	193068
OFF 10	TTTCCATAGAAAACAtTtCAAATgAaA	chr10	105528793	4	0.9	547443	1.0	451210
OFF 11	TTTACATgaAAAACACTGCAAgaAACA	chr6	110538141	4	0.9	637391	0.7	491770
OFF 12	TTTACAaAGAcAcCACTGaAAATAACA	chr9	37009449	4	0.5	211688	0.5	174784

* on-target site

Supplementary Figure 6. Deep sequencing analysis of in silico predicted off-target sites for crRNA+11 in primary airway epithelial cells.

a, Deep sequencing analysis of the *CFTR* on-target locus after AsCas12a-crRNA+11 transduction (15 days) of the 3272-26A>G primary airway cells. **b**, Percentage of deep sequencing reads of the edited and non-edited 3272-26A>G or WT alleles from (**a**). **c**, off-target analysis of in silico predicted sites. All the predicted sites with 4 or less mismatches (12 sites) were analyzed in 3272-26A>G primary airway cells treated as in (**a**).

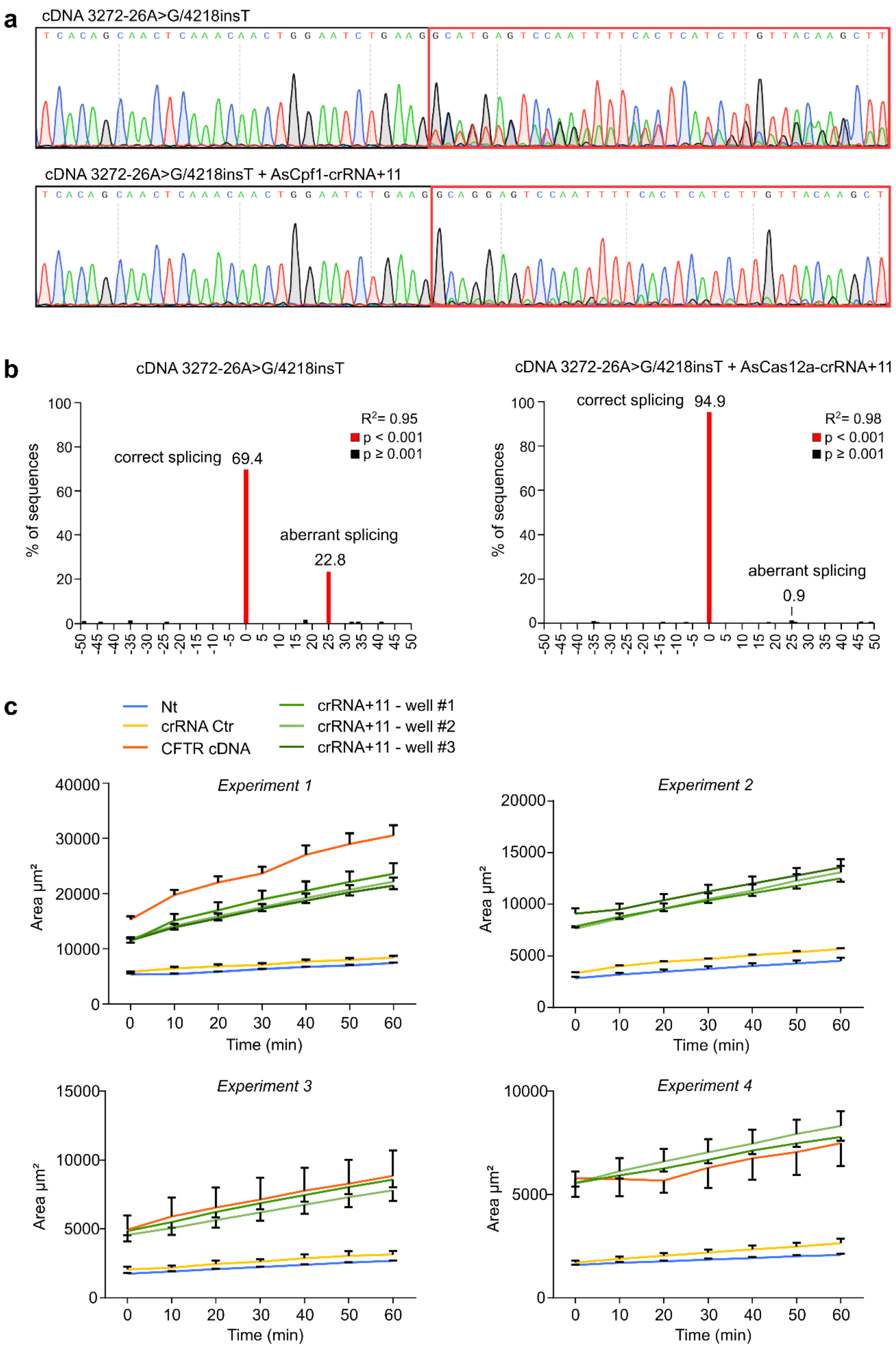
a
cDNA 3272-26A>G/4218insT
cDNA 3272-26A>G/4218insT + AsCpf1-crRNA+11
b
cDNA 3272-26A>G/4218insT
% of sequences
correct splicing 69.4
aberrant splicing
22.8
R2= 0.95
p < 0.001
p ≥ 0.001
cDNA 3272-26A>G/4218insT + AsCas12a-crRNA+11
correct splicing 94.9
aberrant splicing
0.9
R2= 0.98
p < 0.001
p ≥ 0.001
c
Nt
crRNA Ctr
CFTR cDNA
crRNA+11 - well #1
crRNA+11 - well #2
crRNA+11 - well #3
Experiment 1
Experiment 2
Experiment 3
Experiment 4
Area µm²
Time (min)

Supplementary Figure 7. CFTR splicing and functional characterization of 3272-26A>G mutated CF patient's organoids after genome editing with AsCas12a-crRNA+11.

a, Chromatogram of RT-PCR products from Fig. 3c. Upper panel represent the mixed population of mRNA transcripts of 3272-26A>G/4218insT organoids, the lower panel show transcripts after AsCas12a-crRNA+11 editing in these organoids. Red box indicates chromatogram area after exon19-exon20 junction. **b**, Chromatogram deconvolution analysis was used to evaluate the amount of mutated splicing (inclusion of +25 nt from intron 19) before and after AsCas12a-crRNA+11 cleavage. **c**, FIS assay of n=4 independent experiments; each line represents one well (n=25-300). Data are means ± SD.

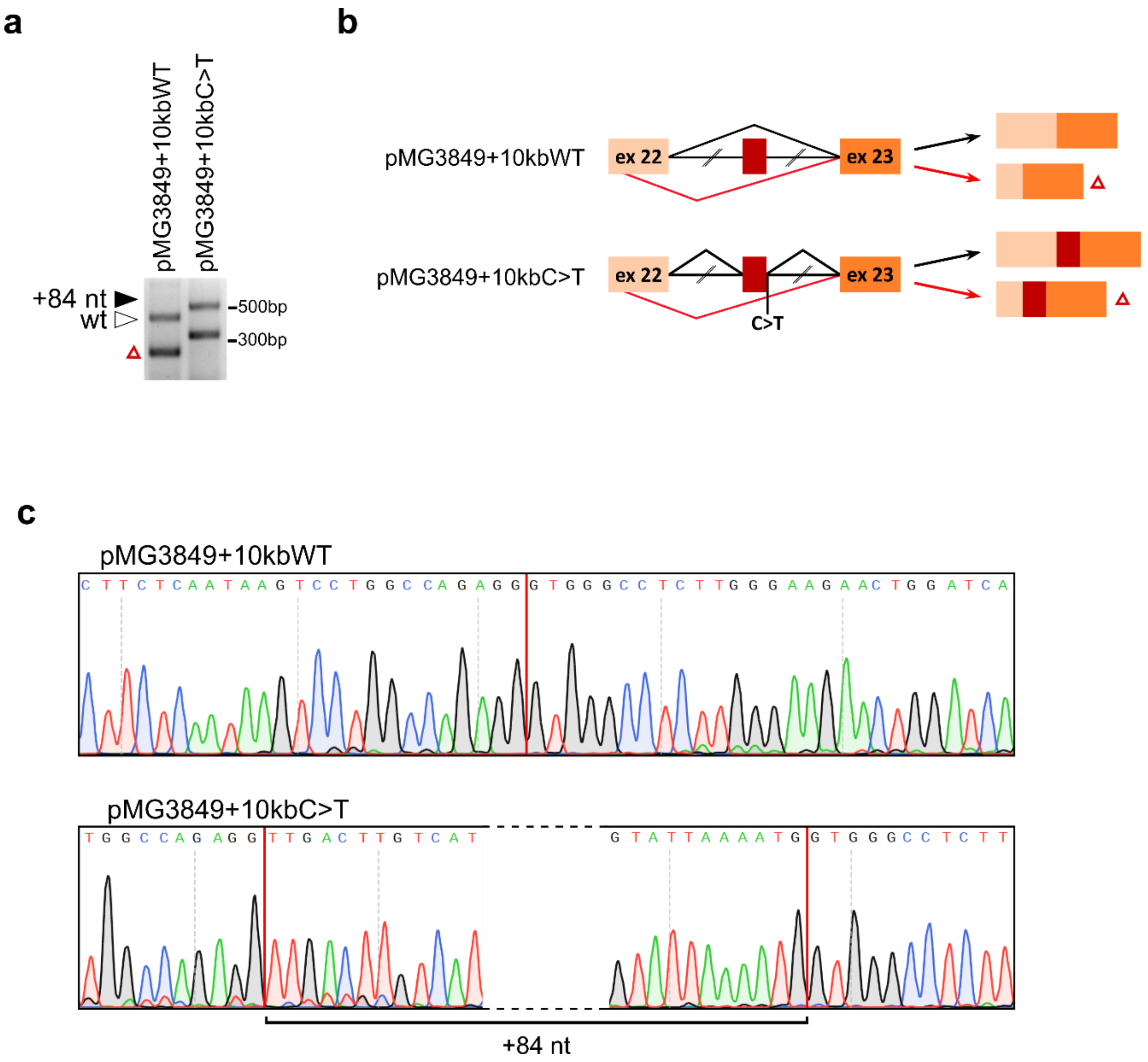

Supplementary Figure 8. Validation of intron 22 splicing in pMG3849+10kbWT and pMG3849+10kbC>T *CFTR* minigene models.

a, Splicing pattern of *CFTR* wild type (pMG3849+10kbWT) and mutated (pMG3849+10kbC>T) minigene models, transfected in HEK293T, by agarose gel electrophoresis analysis of RT-PCR products. Black-solid arrow indicates aberrant splicing, white-empty arrow indicates correct splicing and red triangle indicates a minigene splicing artifact. **b**, Scheme of minigenes splicing products. The minigene splicing artifact was caused by the use of an alternative donor splice site in exon 22, causing the inclusion of only 49 bases of exon 22 into the mature transcripts of the minigene models. **c**, Sanger sequencing chromatogram of minigene splicing products from (**a**). Red lines represent the boundary between exons.

a

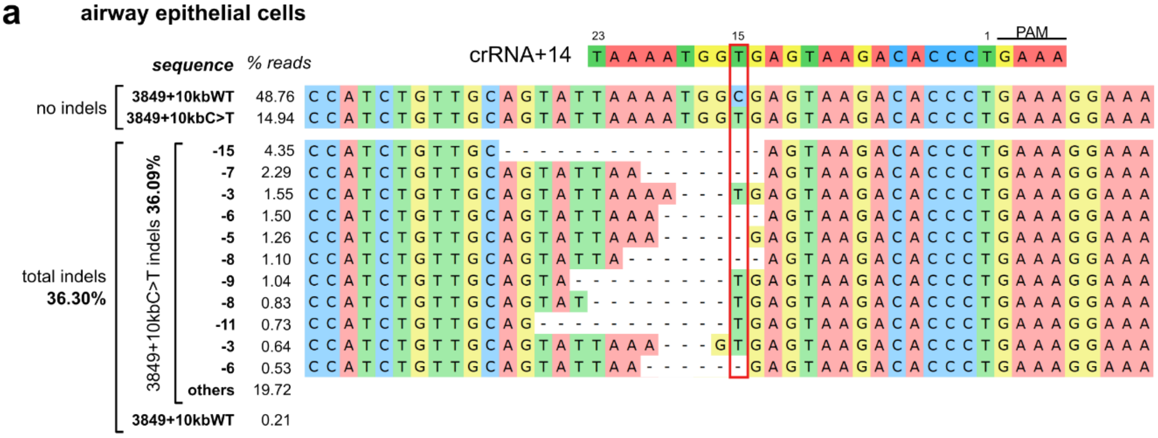

b

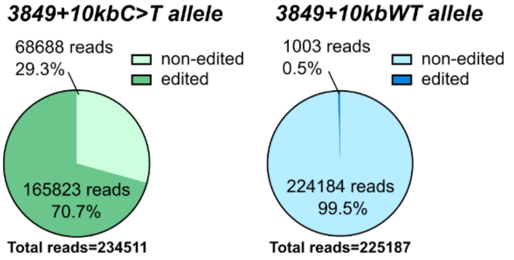

c

AsCas12a-crRNA+14		Chromosome	Position	Mismatches	% editing crRNA+14	total reads crRNA+14	% editing crRNA CTR	total reads crRNA CTR
3849+10kbC>T*	*TTTC*AGGGTGTCTTACTCACCATTTTA	chr7	117639952	0	70.7	234511	0.3	232560
3849+10kbWT	*TTTC*AGGGTGTCTTACTC**g**CCATTTTA	chr7	117639952	1	0.5	225187	0.3	242654
OFF 1	*TTTG*AGGGTGTCTTA**tg**CA**t**CA**a**TTTA	chr4	94977071	4	1.0	485018	0.0	713721
OFF 2	*TTTC*AG**a**GTGTCTTACTCAC**t**ATTT**ct**	chr13	35225035	4	1.0	334723	0.6	448459
OFF 3	*TTTC*AG**a**GT**t**T**ta**TACTCACCATTTTA	chr6	152271172	4	0.5	474836	0.3	564122

* on-target site

d

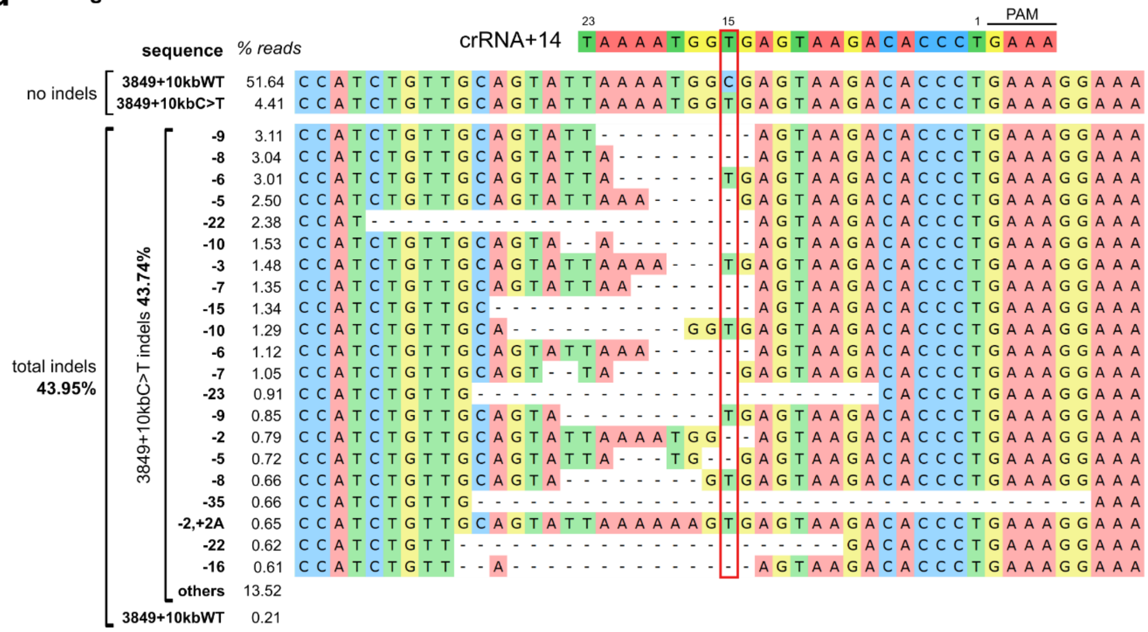

Supplementary Figure 9. Deep sequencing analysis of in silico predicted off-target sites for crRNA+14 in organoids and primary airway epithelial cells.

a, Deep sequencing analysis of the *CFTR* on-target locus after AsCas12a-crRNA+14 transduction (15 days) of the 3849+10kbC>T primary airway cells. **b**, Percentage of deep sequencing reads of the edited and non-edited 3849+10kbC>T or WT alleles in primary airway cells treated as in (**a**). **c**, off-target analysis of in silico predicted sites. All the predicted sites with 4 or less mismatches (3 sites) were analyzed in 3849+10kbC>T primary airway cells treated as in (**b**). **d**, Deep sequencing analysis of the *CFTR* on-target locus after AsCas12a-crRNA+14 transduction (15 days) of the 3849+10kbC>T organoids.

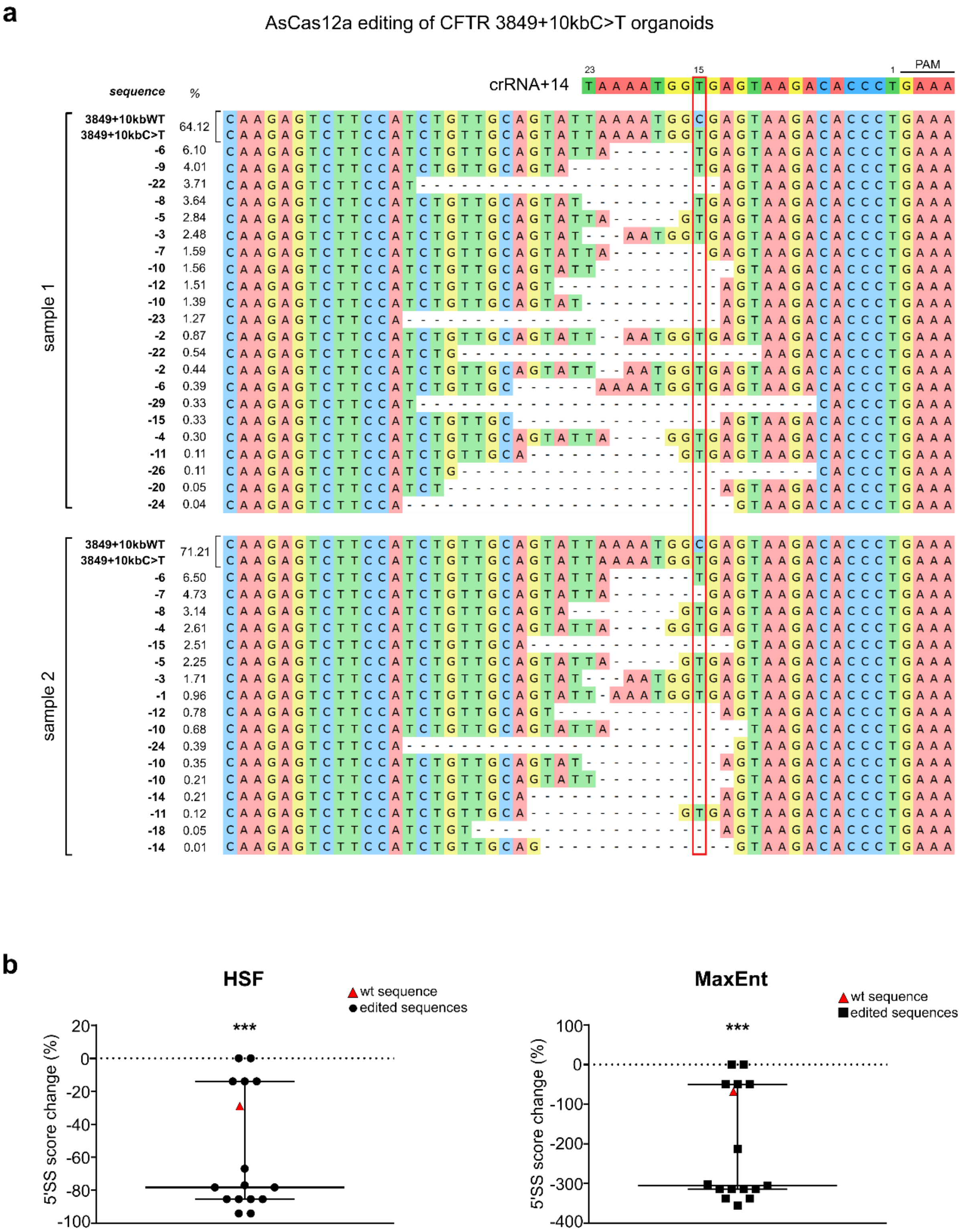
a
AsCas12a editing of CFTR 3849+10kbC>T organoids
crRNA+14
PAM
sequence
%
3849+10kbWT
3849+10kbC>T
sample 1
sample 2
b
HSF
MaxEnt
wt sequence
edited sequences
5'SS score change (%)
***

Supplementary Figure 10. AsCas12a editing of CFTR 3849+10kbC>T organoids and in silico splicing score prediction.

a, SINTHEGO ICE analysis of AsCas12a-crRNA+14 editing in two independent organoids samples. Predicted repair outcomes are represented with their abundance. **b**, Prediction of the score change of AsCas12a-crRNA+14 modification on the mutated 5'splice site (ss) by bioinformatics tools (HSF and MaxEnt prediction algorithms, see Methods section). Data are median with interquartile range. Statistical analysis was performed using two-tailed Wilcoxon signed-rank test; ***$P<0.001$. Score of wild type sequence was given only as reference in the graph.

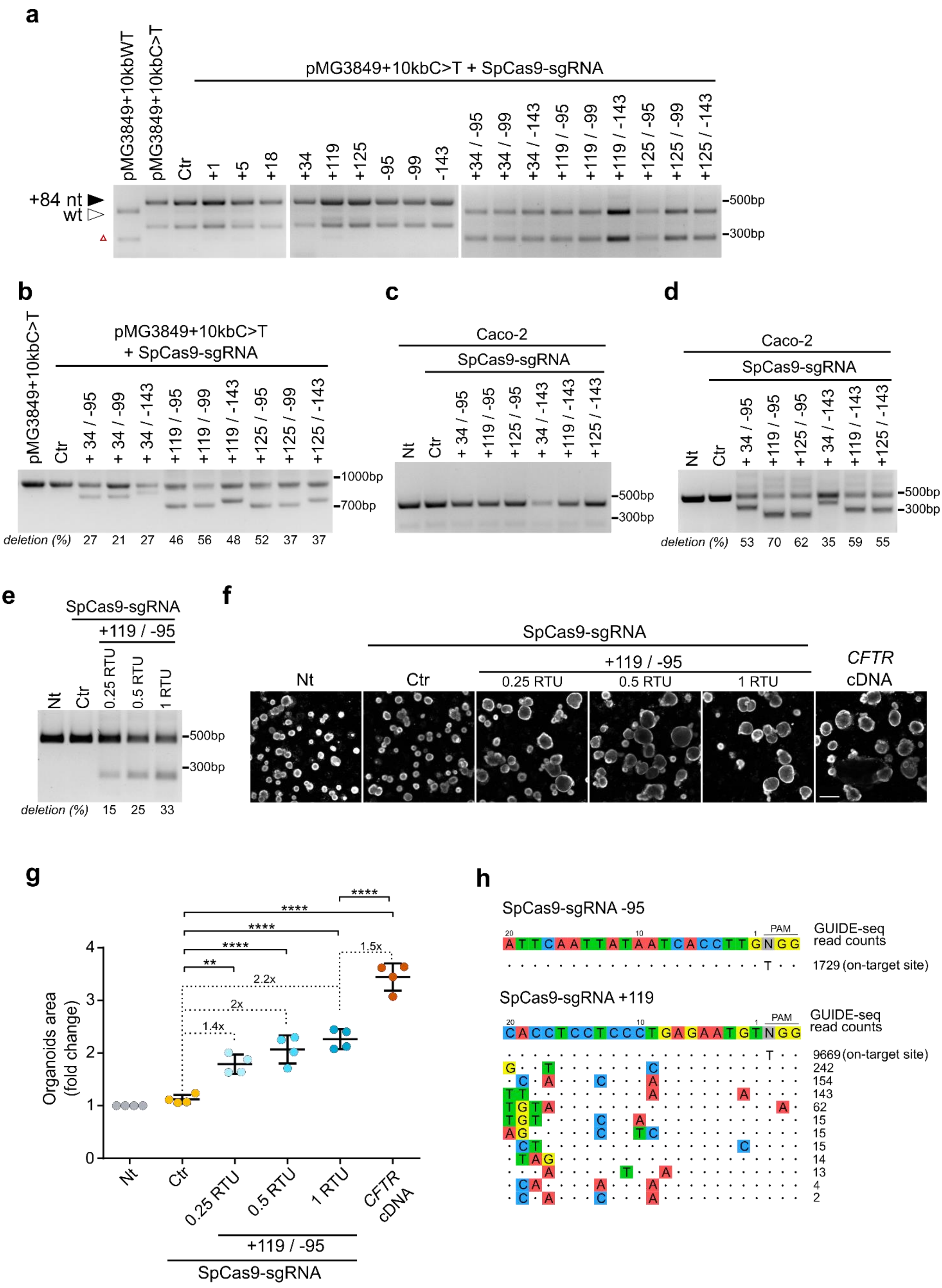
a
pMG3849+10kbWT
pMG3849+10kbC>T
pMG3849+10kbC>T + SpCas9-sgRNA
Ctr
+1
+5
+18
+34
+119
+125
-95
-99
-143
+34 / -95
+34 / -99
+34 / -143
+119 / -95
+119 / -99
+119 / -143
+125 / -95
+125 / -99
+125 / -143
+84 nt
wt
500bp
300bp
b
pMG3849+10kbC>T
+ SpCas9-sgRNA
1000bp
700bp
deletion (%) 27 21 27 46 56 48 52 37 37
c
Caco-2
SpCas9-sgRNA
Nt
d
deletion (%) 53 70 62 35 59 55
e
+119 / -95
0.25 RTU
0.5 RTU
1 RTU
deletion (%) 15 25 33
f
CFTR
cDNA
g
Organoids area
(fold change)
1.4x
2x
2.2x
1.5x
**
****
h
SpCas9-sgRNA -95
PAM
GUIDE-seq
read counts
1729 (on-target site)
SpCas9-sgRNA +119
9669 (on-target site)
242
154
143
62
15
15
15
14
13
4
2

Supplementary Figure 11. SpCas9-sgRNA correction of 3849+10kb splicing defect in a minigene model and CF patient derived intestinal organoids.

a, Screening of SpCas9-sgRNA single or in pairs in pMG3849+10kbC>T transfected in HEK293T cells. RT-PCR products were analyzed by agarose gel electrophoresis. Black-solid arrow indicates aberrant splicing, white-empty arrow indicates correct splicing and red-arrow indicates a minigene splicing artifact. **b**, Agarose gel electrophoretic analysis of targeted deletions in pMG3849+10kbC>T after cleavage of SpCas9-gRNA pairs. **c**, RT-PCR products and **d**, targeted deletions in Caco-2 cells transduced with a SpCas9-sgRNA lentiviral vectors and 10 days of puromycin selection. **e**, Editing in patient organoids analyzed by agarose gel electrophoresis. **f**, Confocal images of calcein labelled CF 3849+10kbC>T organoids at T=0 min transduced with 0.25, 0.5 or 1 RTU of SpCas9-sgRNAs-95/+119. Scale bar = 200 µm. **g**, Quantification of steady-state organoid area; each dot represents the average area of organoids from one well (3-30 organoids per well) from n=1 experiment. Data are means ± SD. Statistical analysis was performed using one-way ANOVA; **P<0.01, ****P<0.0001. **h**, GUIDE-seq analysis of sgRNA-95 and sgRNA+119.
