# Supplementary material for: Allele specific repair of splicing mutations in cystic fibrosis through AsCas12a genome editing
Source: Nat Commun. 2019 Aug 7;10:3556. doi: 10.1038/s41467-019-11454-9 (PMC6685978; doi:10.1038/s41467-019-11454-9)
Supplement: Supplementary file 6 — Data 5 [file 41467_2019_11454_MOESM6_ESM.docx]

pMG3272-26A>GWT

aagcttgTACCATGGGCAAACCAATCCCAAACCCACTGCTGGGTCTGGATggtaccggtgaccttctgcctcTTACCATA

TTTGACTTCATCCAGTTGTTATTAATTGTGATTGGAGCTATAGCAGttgtcgcagtTTTACAACCCTACATCTTTGTTGC

AACAGTGCCAGTGATAGTGGCTTTTATTATGTTGAGAGCATATTTCCTCCAAACCTCACAGCAACTCAAACAACTGGAAT

CTGAAGGTATGACAGTGAATGTGCGATACTCATCTTGTAAAAAAGCTATAAGAGCTATTTGAGATTCTTTATTGTTAATC

TACTTAAAAAAAATTCTGCTTTTAAACTTTTACATCATATAACAATAATTTTTTTCTACATGCATGTGTATATAAAAGGA

AACTATATTACAAAGTACACATGGATTTTTTTTCTTAATTAATGACCATGTGACTTCATTTTGGTTTTAAAATAGGTATA

TAGAATCTTACCACAGTTGGTGTACAGGACATTCATTTATAATAAACTTATATCAGTCAAATTAAACAAGGATAGTGCTG

CTATTACTAAAGGTTTCTCTGGGTTCCCAAATGATACTTGACCAAATTTGTCCCTTTGGCTTGTTGTCTTCAGACACCCT

TTCTTCATGTGTTGGAGCTGCCATTTCGTGTGCCCCCAAACTCTACTTGAGCTGTTAGGGAATCACATTTTGCAGTGACA

GCCTTAGTGTGGGTGCATTTTCAGGCAATACTTTTTCAGTATATTTCTGCTTTGTAGATTATTAGCTAAATCAAGTCACA

TAAACTTCCTTAATTTAGATACTTGAAAAAATTGTCTTAAAAGAAAATTTTTTTAGTAAGAATTAATTTAGAATTAGCCA

GAAAACTCCCAGTGGTAGCCAAGAAAGAGGAATAAATATTGGTGGTAATTTTTTAAGTTCCCATCTCTGGTAGCCAAGTA

AAAAAAGAGGGTAACTCATTAATAAAATAACAAATCATATCTATTCAAAGAATGGCACCAGTGTGAAAAAAAGCTTTTTA

ACCAATGACATTTGTGATATGATTATTCTAATTTAGTCTTTTTCAGGTACAAGATATTATGAAATTACATTTTGTGTTTA

TGTTATTTGCAaTGTTTTCTATGGAAATATTTCACagGCAGGAGTCCAATTTTCACTCATCTTGTTACAAGCTTAAAAGG

ACTATGGACACTTCGTGCCTTCGGACGGCAGCCTTACTTTGAAACTCTGTTCCACAAAGCTCTGAATTTACATACTGCCA

ACTGGTTCTTGTACCTGTCAACACTGCGCTGGTTCCAAATGAGAATAGAAATGATTTTTGTCATCTTCTTCATTGCTGTT

ACCTTCATTTCCATTTTAACAACAGgcgaggGATCCggAGAgAATTTATAcTTcCAGGGCgggAATTCgggcTCCATTTC

TTCTAGTATCTTTAAAAATGAAGGTtaagcggccgctctaga

pMG3272-26A>G

aagcttgTACCATGGGCAAACCAATCCCAAACCCACTGCTGGGTCTGGATggtaccggtgaccttctgcctcTTACCATA

TTTGACTTCATCCAGTTGTTATTAATTGTGATTGGAGCTATAGCAGttgtcgcagtTTTACAACCCTACATCTTTGTTGC

AACAGTGCCAGTGATAGTGGCTTTTATTATGTTGAGAGCATATTTCCTCCAAACCTCACAGCAACTCAAACAACTGGAAT

CTGAAGGTATGACAGTGAATGTGCGATACTCATCTTGTAAAAAAGCTATAAGAGCTATTTGAGATTCTTTATTGTTAATC

TACTTAAAAAAAATTCTGCTTTTAAACTTTTACATCATATAACAATAATTTTTTTCTACATGCATGTGTATATAAAAGGA

AACTATATTACAAAGTACACATGGATTTTTTTTCTTAATTAATGACCATGTGACTTCATTTTGGTTTTAAAATAGGTATA

TAGAATCTTACCACAGTTGGTGTACAGGACATTCATTTATAATAAACTTATATCAGTCAAATTAAACAAGGATAGTGCTG

CTATTACTAAAGGTTTCTCTGGGTTCCCAAATGATACTTGACCAAATTTGTCCCTTTGGCTTGTTGTCTTCAGACACCCT

TTCTTCATGTGTTGGAGCTGCCATTTCGTGTGCCCCCAAACTCTACTTGAGCTGTTAGGGAATCACATTTTGCAGTGACA

GCCTTAGTGTGGGTGCATTTTCAGGCAATACTTTTTCAGTATATTTCTGCTTTGTAGATTATTAGCTAAATCAAGTCACA

TAAACTTCCTTAATTTAGATACTTGAAAAAATTGTCTTAAAAGAAAATTTTTTTAGTAAGAATTAATTTAGAATTAGCCA

GAAAACTCCCAGTGGTAGCCAAGAAAGAGGAATAAATATTGGTGGTAATTTTTTAAGTTCCCATCTCTGGTAGCCAAGTA

AAAAAAGAGGGTAACTCATTAATAAAATAACAAATCATATCTATTCAAAGAATGGCACCAGTGTGAAAAAAAGCTTTTTA

ACCAATGACATTTGTGATATGATTATTCTAATTTAGTCTTTTTCAGGTACAAGATATTATGAAATTACATTTTGTGTTTA

TGTTATTTGCAgTGTTTTCTATGGAAATATTTCACagGCAGGAGTCCAATTTTCACTCATCTTGTTACAAGCTTAAAAGG

ACTATGGACACTTCGTGCCTTCGGACGGCAGCCTTACTTTGAAACTCTGTTCCACAAAGCTCTGAATTTACATACTGCCA

ACTGGTTCTTGTACCTGTCAACACTGCGCTGGTTCCAAATGAGAATAGAAATGATTTTTGTCATCTTCTTCATTGCTGTT

ACCTTCATTTCCATTTTAACAACAGgcgaggGATCCggAGAgAATTTATAcTTcCAGGGCgggAATTCgggcTCCATTTC

TTCTAGTATCTTTAAAAATGAAGGTtaagcggccgctctaga

pMG3849+10kbWT

TACTTAATACGACTCACTATAGGCTAGCCtcgagATGCGATCTGTGAGCCGAGTCTTTAAGTTCATTGACATGCCAACAG

AAGGTAAACCTACCAAGTCAACCAAACCATACAAGAATGGCCAACTCTCGAAAGTTATGATTATTGAGAATTCACACGTG

AAGAAAGATGACATCTGGCCCTCAGGGGGCCAAATGACTGTCAAAGATCTCACAGCAAAATACACAGAAGGTGGAAATGC

CATATTAGAGAACATTTCCTTCTCAATAAGTCCTGGCCAGAGGGTGAGATTTGAACACTGCTTGCTTTGTTAGACTGTGT

TCAGTAAGTGAATCCCAGTAGCCTGAAGCAATGTGTTAGCAGAATCTATTTGTAACATTATTATTGTACAGTAGAATCAA

TATTAAACACACATGTTTTATTATATGGAGTCATTATTTTTAATATGAAATTTAATTTGCAGAGTCCTGAACCTATATAT

TCAGTGGGTATAAGCAGCATATTCTCAATACTATGTTTCATTAATAATTAATAGAGATATATGAACACATAAAAGATTCA

ATTATAATCACCTTGTGGATCTAAATTTCAGTTGACTTGTCATCTTGATTTCTGGAGACCACAAGGTAATGAAAAATAAT

TACAAGAGTCTTCCATCTGTTGCAGTATTAAAATGGCGAGTAAGACACCCTGAAAGGAAATGTTCTATTCATGGTACAAT

GCAATTACAGCTAGCACCAAATTCAACACTGTTTAACTTTCAACATATTATTTTGATTTATCTTGATCCAACATTCTCAG

GGAGGAGGTGCATTGAAGTTATTAGAAAACACTGACTTAGATTTAGGGTATGTCTTAAAAGCTTATTTGCGGGAAGTACT

CTAGCCTTATTCAACAGATCACTGAGAAGCCTAAAGGTCAGTGATAAAGGAAGTCTGCATCAGGGGTCCAATTCCTTATG

GCCAGTTTCTCTATTCTGTTCCAAGGTTGTTTGTCTCCATATATCAACATTGGTCAGGATTGAAAGTGTGCAACAAGGTT

TGAATGAATAAGTGAAAATCTTCCACTGGTGACAGGATAAAATATTCCAATGGTTTTTATTGAAGTACAATACTGAATTA

TGTTTATGGCATGGTACCTATATGTCACAGAAGTGATCCCATCACTTTTACCTTATAGGTGGGCCTCTTGGGAAGAACTG

GATCAGGGAAGAGTACTTTGTTATCAGCTTTTTTGAGACTACTGAACACTGAAGGAGAAATCCAGATCGATGGTGTGTCT

TGGGATTCAATAACTTTGCAACAGTGGAGGAAAGCCTTTGGAGTGATACCACAGgcggccgcT

pMG3849+10kbC>T

TACTTAATACGACTCACTATAGGCTAGCCtcgagATGCGATCTGTGAGCCGAGTCTTTAAGTTCATTGACATGCCAACAG

AAGGTAAACCTACCAAGTCAACCAAACCATACAAGAATGGCCAACTCTCGAAAGTTATGATTATTGAGAATTCACACGTG

AAGAAAGATGACATCTGGCCCTCAGGGGGCCAAATGACTGTCAAAGATCTCACAGCAAAATACACAGAAGGTGGAAATGC

CATATTAGAGAACATTTCCTTCTCAATAAGTCCTGGCCAGAGGGTGAGATTTGAACACTGCTTGCTTTGTTAGACTGTGT

TCAGTAAGTGAATCCCAGTAGCCTGAAGCAATGTGTTAGCAGAATCTATTTGTAACATTATTATTGTACAGTAGAATCAA

TATTAAACACACATGTTTTATTATATGGAGTCATTATTTTTAATATGAAATTTAATTTGCAGAGTCCTGAACCTATATAT

TCAGTGGGTATAAGCAGCATATTCTCAATACTATGTTTCATTAATAATTAATAGAGATATATGAACACATAAAAGATTCA

ATTATAATCACCTTGTGGATCTAAATTTCAGTTGACTTGTCATCTTGATTTCTGGAGACCACAAGGTAATGAAAAATAAT

TACAAGAGTCTTCCATCTGTTGCAGTATTAAAATGGtGAGTAAGACACCCTGAAAGGAAATGTTCTATTCATGGTACAAT

GCAATTACAGCTAGCACCAAATTCAACACTGTTTAACTTTCAACATATTATTTTGATTTATCTTGATCCAACATTCTCAG

GGAGGAGGTGCATTGAAGTTATTAGAAAACACTGACTTAGATTTAGGGTATGTCTTAAAAGCTTATTTGCGGGAAGTACT

CTAGCCTTATTCAACAGATCACTGAGAAGCCTAAAGGTCAGTGATAAAGGAAGTCTGCATCAGGGGTCCAATTCCTTATG

GCCAGTTTCTCTATTCTGTTCCAAGGTTGTTTGTCTCCATATATCAACATTGGTCAGGATTGAAAGTGTGCAACAAGGTT

TGAATGAATAAGTGAAAATCTTCCACTGGTGACAGGATAAAATATTCCAATGGTTTTTATTGAAGTACAATACTGAATTA

TGTTTATGGCATGGTACCTATATGTCACAGAAGTGATCCCATCACTTTTACCTTATAGGTGGGCCTCTTGGGAAGAACTG

GATCAGGGAAGAGTACTTTGTTATCAGCTTTTTTGAGACTACTGAACACTGAAGGAGAAATCCAGATCGATGGTGTGTCT

TGGGATTCAATAACTTTGCAACAGTGGAGGAAAGCCTTTGGAGTGATACCACAGgcggccgc

cDNA pMG3849+10kbWT - splicing artifact

ATGCGATCTGTGAGCCGAGTCTTTAAGTTCATTGACATGCCAACAGAAGGTGGGCCTCTTGGGAAGAACTGGATCAGGGAAGAGTACTTTGTTATCAGCTTTTTTGAGACTACTGAACACTGAAGGAGAAATCCAGATCGATGGTGTGTCTTGGGATTCAATAACTTTGCAACAGTGGAGGAAAGCCTTTGGAGTGATACCACAG

cDNA pMG3849+10kbC>T - splicing artifact

ATGCGATCTGTGAGCCGAGTCTTTAAGTTCATTGACATGCCAACAGAAGTTGACTTGTCATCTTGATTTCTGGAGACCACAAGGTAATGAAAAATAATTACAAGAGTCTTCCATCTGTTGCAGTATTAAAATGGTGGGCCTCTTGGGAAGAACTGGATCAGGGAAGAGTACTTTGTTATCAGCTTTTTTGAGACTACTGAACACTGAAGGAGAAATCCAGATCGATGGTGTGTCTTGGGATTCAATAACTTTGCAACAGTGGAGGAAAGCCTTTGGAGTGATACCACAG
