# Supplementary material for: Allele specific repair of splicing mutations in cystic fibrosis through AsCas12a genome editing
Source: Nat Commun. 2019 Aug 7;10:3556. doi: 10.1038/s41467-019-11454-9 (PMC6685978; doi:10.1038/s41467-019-11454-9)
Supplement: Supplementary file 8 — Reporting Summary [file 41467_2019_11454_MOESM8_ESM.pdf]

natureresearch

Corresponding author(s): Anna Cereseto, Marianne Carlon, Gianluca Petris

Last updated by author(s): Jun 25, 2019

Reporting Summary

Nature Research wishes to improve the reproducibility of the work that we publish. This form provides structure for consistency and transparency in reporting. For further information on Nature Research policies, see Authors & Referees and the Editorial Policy Checklist.

Statistics

For all statistical analyses, confirm that the following items are present in the figure legend, table legend, main text, or Methods section.

n/a	Confirmed	
☐	☒	The exact sample size (*n*) for each experimental group/condition, given as a discrete number and unit of measurement
☐	☒	A statement on whether measurements were taken from distinct samples or whether the same sample was measured repeatedly
☐	☒	The statistical test(s) used AND whether they are one- or two-sided<br>*Only common tests should be described solely by name; describe more complex techniques in the Methods section.*
☐	☒	A description of all covariates tested
☐	☒	A description of any assumptions or corrections, such as tests of normality and adjustment for multiple comparisons
☐	☒	A full description of the statistical parameters including central tendency (e.g. means) or other basic estimates (e.g. regression coefficient) AND variation (e.g. standard deviation) or associated estimates of uncertainty (e.g. confidence intervals)
☐	☒	For null hypothesis testing, the test statistic (e.g. *F*, *t*, *r*) with confidence intervals, effect sizes, degrees of freedom and *P* value noted<br>*Give P values as exact values whenever suitable.*
☒	☐	For Bayesian analysis, information on the choice of priors and Markov chain Monte Carlo settings
☒	☐	For hierarchical and complex designs, identification of the appropriate level for tests and full reporting of outcomes
☒	☐	Estimates of effect sizes (e.g. Cohen's *d*, Pearson's *r*), indicating how they were calculated

Our web collection on statistics for biologists contains articles on many of the points above.

Software and code

Policy information about availability of computer code

Data collection	Zen Blue software, version 2.3
Data analysis	Excel<br>GraphPad Prism version 6<br>ImageJ software<br>Alliance UVITEC<br>Human Splicing Finder website (www.umd.be/HSF3/)<br>TIDE online software (https://www.deskgen.com/landing/tide.html)<br>SYNTHEGO ICE online software (https://ice.synthego.com/#/)<br>CRISPResso online software (http://crispresso.pinellolab.partners.org/)

For manuscripts utilizing custom algorithms or software that are central to the research but not yet described in published literature, software must be made available to editors/reviewers. We strongly encourage code deposition in a community repository (e.g. GitHub). See the Nature Research guidelines for submitting code & software for further information.

Data

Policy information about availability of data

All manuscripts must include a data availability statement. This statement should provide the following information, where applicable:

- Accession codes, unique identifiers, or web links for publicly available datasets
- A list of figures that have associated raw data
- A description of any restrictions on data availability

All data associated with this study are present in the paper, in the Supplementary Materials or are available from the authors upon reasonable request. All materials can be requested via A.C., M.S.C. or G.P.

Field-specific reporting

Please select the one below that is the best fit for your research. If you are not sure, read the appropriate sections before making your selection.

☒ Life sciences ☐ Behavioural & social sciences ☐ Ecological, evolutionary & environmental sciences

For a reference copy of the document with all sections, see nature.com/documents/nr-reporting-summary-flat.pdf

Life sciences study design

All studies must disclose on these points even when the disclosure is negative.

Sample size	2 or more experiments were performed using HEK293 or primary airway epithelial cells and human intestinal organoids. Experiments in human cells were performed in samples derived from 4 different patients.
Data exclusions	Some experiments were excluded based on technical grounds when it was evident from internal control conditions that cells were not functioning. For organoids analysis, defective particles with an area less than 1500 or 3000 µm for 3272-26A>G or 3849+10Kb C>T, respectively, were excluded from the analysis.
Replication	Experiments were considered reproducible if at least 2 replicates analysis produced similar results.
Randomization	Not relevant because each experiment involved its own control and test contions
Blinding	Blinding is not a standard practice for the type of experiments described in the paper.

Reporting for specific materials, systems and methods

We require information from authors about some types of materials, experimental systems and methods used in many studies. Here, indicate whether each material, system or method listed is relevant to your study. If you are not sure if a list item applies to your research, read the appropriate section before selecting a response.

Materials & experimental systems

n/a	Involved in the study	
☒	☐	Antibodies
☐	☒	Eukaryotic cell lines
☒	☐	Palaeontology
☒	☐	Animals and other organisms
☒	☐	Human research participants
☒	☐	Clinical data

Methods

n/a	Involved in the study	
☒	☐	ChIP-seq
☒	☐	Flow cytometry
☒	☐	MRI-based neuroimaging

Eukaryotic cell lines

Policy information about cell lines

Cell line source(s)	HEK293T, HEK293 and Caco-2 cells were obtained from American Type Culture Collection (ATCC; www.atcc.org). Primary airway epithelial cells were obtained from Italian Cystic Fibrosis Research Foundation. Human intestinal organoids were obtained from CF Centre, Organ System Unit, KU Leuven.
Authentication	ATCC deposited cell lines.
Mycoplasma contamination	All the cells were tested for mycoplasma contamination. All samples resulted negative for the test.
Commonly misidentified lines (See ICLAC register)	None
