# Supplementary material for: Allele specific repair of splicing mutations in cystic fibrosis through AsCas12a genome editing
Source: Nat Commun. 2019 Aug 7;10:3556. doi: 10.1038/s41467-019-11454-9 (PMC6685978; doi:10.1038/s41467-019-11454-9)
Supplement: Supplementary file 9 — Description of Additional Supplementary Files [file 41467_2019_11454_MOESM9_ESM.docx]

**Title:** Supplementary Data 1.
**Description:** List of AsCas12a and SpCas9 gRNAs.

**Title:** Supplementary Data 2.
**Description:** List of AsCas12a-crRNA+11 indels in 3272-26A>G CF patientderived organoids from deep sequencing data.

**Title:** Supplementary Data 3.
**Description:** GUIDE-seq data.

**Title:** Supplementary Data 4.
**Description:** List of oligonucleotides used for PCR.

**Title:** Supplementary Data 5.
**Description:** Minigene plasmid sequences.

**Title:** Supplementary Data 6.
**Description:** In silico splicing score prediction.
